# Replication stress conferred by POT1 dysfunction promotes telomere relocalization to the nuclear pore

**DOI:** 10.1101/gad.337287.120

**Published:** 2020-12-01

**Authors:** Alexandra M. Pinzaru, Mike Kareh, Noa Lamm, Eros Lazzerini-Denchi, Anthony J. Cesare, Agnel Sfeir

**Affiliations:** 1 Skirball Institute of Biomolecular Medicine, Department of Cell Biology, New York University School of Medicine, New York, New York 10016, USA;; 2 Children's Medical Research Institute, University of Sydney, Westmead, New South Wales 2145, Australia;; 3 National Cancer Institute, National Institutes of Health, Bethesda, Maryland 20892, USA

**Keywords:** POT1, telomeres, replication stress, nuclear pore, nuclear F-actin, nuclear periphery, MiDAS, CRISPRi

## Abstract

Mutations in the telomere-binding protein POT1 are associated with solid tumors and leukemias. POT1 alterations cause rapid telomere elongation, ATR kinase activation, telomere fragility, and accelerated tumor development. Here, we define the impact of mutant *POT1* alleles through complementary genetic and proteomic approaches based on CRISPR interference and biotin-based proximity labeling, respectively. These screens reveal that replication stress is a major vulnerability in cells expressing mutant POT1, which manifests as increased telomere mitotic DNA synthesis at telomeres. Our study also unveils a role for the nuclear pore complex in resolving replication defects at telomeres. Depletion of nuclear pore complex subunits in the context of POT1 dysfunction increases DNA damage signaling, telomere fragility and sister chromatid exchanges. Furthermore, we observed telomere repositioning to the nuclear periphery driven by nuclear F-actin polymerization in cells with *POT1* mutations. In conclusion, our study establishes that relocalization of dysfunctional telomeres to the nuclear periphery is critical to preserve telomere repeat integrity.

## Abstract (precis)

In this study, Pinzaru et al. set out to uncover the pathways that enable the proliferation of cells expressing cancer-associated POT1 mutations. Using complementary genetic and proteomic approaches, the authors identify a conserved function for the NPC in resolving replication defects at telomere loci.

Mammalian telomeres are composed of tracts of duplex TTAGGG repeats that are extended by the telomerase reverse transcriptase (Greider and Blackburn 1985). Telomere repeats are bound by a six-subunit complex, termed shelterin, that regulates DNA damage response and repair activities at chromosome ends (de Lange 2005). Shelterin is composed of TRF1 and TRF2 that bind to the duplex region of the telomeres and recruit RAP1, TIN2, TPP1, and the single-strand DNA (ssDNA)-binding protein, POT1.

Protection of telomeres 1 (POT1) contains two N-terminal oligonucleotide/oligosaccharide binding (OB) fold domains that bind to the ssDNA (T)TAGGGTTAG sequence present at the 3′ overhang or internally within exposed segments of the telomere (Baumann and Cech 2001; Lei et al. 2004). Despite its subnanomolar affinity to ssDNA, POT1 relies on its interaction with TPP1 for telomere recruitment (Liu et al. 2004; Hockemeyer et al. 2007; Nandakumar et al. 2010), and the POT1-TPP1 heterodimer is anchored to the rest of the shelterin complex through TIN2 (Liu et al. 2004; Ye et al. 2004; Takai et al. 2011). POT1 binding to the 3′ overhang occludes telomerase from accessing its substrate and negatively regulates telomere length (Loayza and De Lange 2003). POT1 also plays a key role in telomere protection. Depleting POT1 in human cells induces telomere DNA damage response signaling (Veldman et al. 2004; Hockemeyer et al. 2005). Rodent telomeres contain two closely related POT1 proteins, POT1a and POT1b, and simultaneous deletion of both genes leads to telomere RPA loading, ATR signaling, and reduced cellular proliferation (Hockemeyer et al. 2006; Wu et al. 2006; Denchi and de Lange 2007; Gong and de Lange 2010).

Cancer genome sequencing identified recurrent *POT1* mutations in tumors from multiple tissue types. Sporadic missense and nonsense mutations were first reported in chronic lymphocytic leukemia (CLL) (Quesada et al. 2012; Ramsay et al. 2013) and later found in parathyroid adenoma (Newey et al. 2012), mantle cell lymphoma (Zhang et al. 2014), and adult T-cell leukemia/lymphoma (Kataoka et al. 2015). Additionally, *POT1* germline mutations were associated with familial cancers, including melanoma (Robles-Espinoza et al. 2014; Shi et al. 2014), and glioma (Bainbridge et al. 2015). *POT1* oncogenic mutations are enriched within the OB fold domains and many alterations disrupt POT1 binding to telomere ssDNA in vitro (Ramsay et al. 2013; Robles-Espinoza et al. 2014; Pinzaru et al. 2016). Consistent with cancer-associated *POT1* mutations causing telomere deprotection, expression of *POT1* OB-fold mutations in human cells results in rapid telomere elongation, telomere fragility, and ATR activation (Ramsay et al. 2013; Shi et al. 2014; Calvete et al. 2015; Pinzaru et al. 2016).

Due to its G-rich sequence, telomeric DNA is prone to forming stable secondary structures (Parkinson et al. 2002) that can impede replication fork progression and promote telomere fragility (Martinez et al. 2009; Sfeir et al. 2009). Several telomere-associated factors assist in the synthesis of telomere DNA to prevent replication stress. For example, the shelterin subunit TRF1 recruits two helicases, RTEL1 and BLM, to unwind secondary structures and prevent replisome stalling in the telomeric DNA (Sfeir et al. 2009; Vannier et al. 2012; Zimmermann et al. 2014). The polymerase-α primase accessory complex, CST (CTC1–STN1–TEN1), can also counteract fork stalling by facilitating repriming of DNA synthesis (Stewart et al. 2012; Wang et al. 2012; Kasbek et al. 2013). DNA combing analyses revealed that telomeres frequently undergo fork stalling events in cells expressing mutant POT1 (Pinzaru et al. 2016). While the mechanistic basis by which *POT1* mutations compromise fork progression remains unknown, genetic evidence suggests that *POT1* mutations act in the same pathway as the CST complex (Pinzaru et al. 2016).

Studies in *S. cerevisiae* revealed that eroded telomeres are targeted to the nuclear pore complex (NPC) (Khadaroo et al. 2009). Enrichment of yeast telomeres at the NPC is driven by RPA SUMOylation by Slx5–Slx8 (Churikov et al. 2016) and enhances the formation of type II survivors (Khadaroo et al. 2009). In mammalian cells, telomeres are transiently tethered to the nuclear envelope during postmitotic nuclear reassembly (Crabbe et al. 2012). Notably, increased mobility of dysfunctional telomeres was observed in response to TRF2 deletion and was mediated by the linker of nucleoskeleton and cytoskeleton complex (Lottersberger et al. 2015). Induction of telomere-specific double-strand breaks (DSBs) using the FokI endonuclease triggered directional mobility and RAD51-dependent telomere clustering in U2OS cells (Cho et al. 2014). In both cases, telomere repositioning to the nuclear periphery was not reported. Furthermore, these studies examined the impact of telomere DSBs and ATM-dependent telomere DDR activation on telomere mobility, and thus the effect of replication defects on mammalian telomere relocalization remains unknown.

Here we aimed to uncover the pathways that enable the proliferation of cells expressing cancer-associated *POT1* mutations. To do so, we performed a CRISPR interference genetic screen alongside a proximity ligation-based proteomic approach. These complementary efforts identified several synthetic lethal pathways that underscored replication stress as a vulnerability associated with POT1 dysfunction. Consistent with unresolved replication problems, we detected an increase in the frequency of mitotic DNA synthesis (MiDAS) at telomeres when POT1 was impaired. In addition, we uncovered a role for the NPC in resolving telomere replication defects in mammalian cells and observed F-actin-dependent accumulation of telomeres at the nuclear periphery in *POT1* mutant cells. In summary, our study unveils a conserved function for the NPC in resolving replication defects at telomere loci from yeast to man.

## Results

### Genome-wide CRISPR interference screen identifies synthetic lethal interactions in cells expressing mutant POT1

To uncover genetic vulnerabilities in cells expressing pathogenic *POT1* mutations, we performed a synthetic lethal (SL) screen in human cells transduced with wild-type POT1 (POT1-WT), POT1-ΔOB, and POT1-K90E (Supplemental Fig. S1A,B). POT1-ΔOB lacks the first OB fold and served as a surrogate for cancer-associated POT1 variants that cannot bind to ssDNA (Loayza and De Lange 2003; Hockemeyer et al. 2007; Calvete et al. 2015). The K90E substitution did not prevent DNA binding in vitro (Pinzaru et al. 2016) but was repeatedly identified in somatic and familial cancers (Quesada et al. 2012; Wilson et al. 2017) and triggered mild telomere dysfunction (Pinzaru et al. 2016). Both alleles retained functional interaction with TPP1 and were efficiently recruited to telomere chromatin (Loayza and De Lange 2003; Pinzaru et al. 2016).

**Figure 1.**
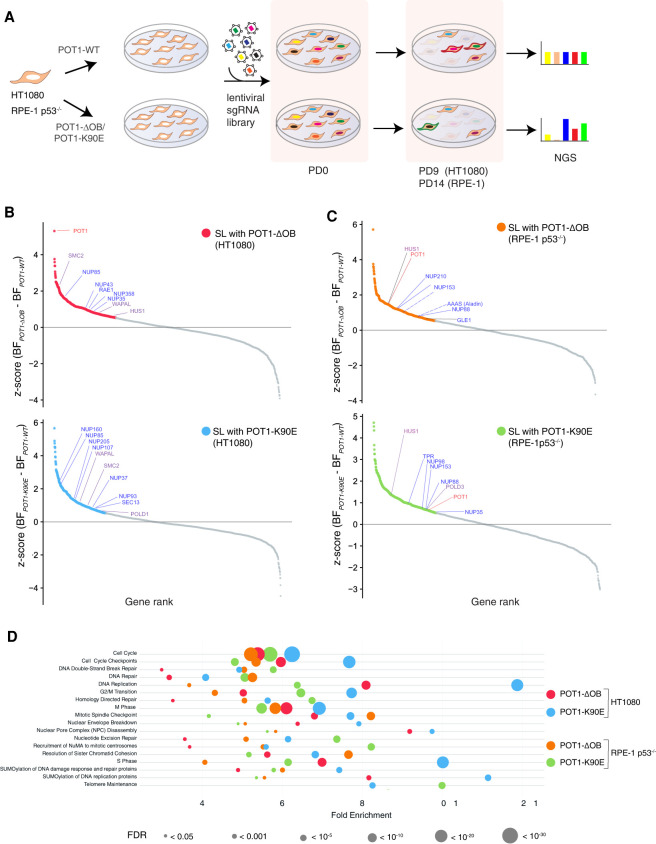
CRISPR interference screens identify synthetic lethalities in cells expressing *POT1* mutations. (*A*) Schematic of the CRIPSRi screening pipeline. HT1080 and RPE-1 p53^−/−^ cells stably expressing dCas9-KRAB, as well as POT1-WT, POT1-ΔOB, and POT1-K90E, were transduced with the genome-wide hCRISPRi v2.1 sgRNA library (Horlbeck et al. 2016). Cells were collected following the selection of sgRNA-expressing cells and designated population doubling (PD) 0. Cells were also collected after approximately nine PDs and ∼14 PDs for HT1080 and RPE-1 p53^−/−^ cells, respectively. The relative change in sgRNA abundance at the time of final collection compared with PD0 was determined by next-generation sequencing (NGS). (*B*) Ranked z-scores of the difference in Bayes factor (BF) scores for “essential” genes in POT1-ΔOB versus POT1-WT (*top*) and POT1-K90E *vs.* POT1-WT (*bottom*) in HT1080 cells. BF scores were determined using the BAGEL analysis pipeline (Hart and Moffat 2016). Genes with a *z*-score ≥0.53 were considered to be potentially synthetic lethal (SL) with mutant POT1. SL candidates are marked in red for POT1-ΔOB and in blue for POT1-K90E. (*C*) Similar analysis as in *B* for RPE-1 p53^−/−^ cells. SL candidates are marked in orange for POT1-ΔOB and in green for POT1-K90E. (*B*,*C*) Selected SL candidate genes highlighted in blue depict nucleoporins (NUPs), purple denotes mitotic DNA synthesis (MiDAS), and general replication stress-related genes, and POT1 is highlighted in red. (*D*) Reactome pathway overrepresentation analysis of synthetic lethal genes with mutant POT1. The analysis was performed using PANTHER classification (v. 14.1). Fold enrichment of each pathway is plotted on the *X*-axis. The false discovery rate (FDR) associated with the fold enrichment is indicated by the size of the circle (the lower the FDR, the larger the circle size).

We performed genome-wide loss-of-function screens in human fibrosarcoma HT1080 cells using CRISPR interference (CRISPRi) (Gilbert et al. 2014; Horlbeck et al. 2016). Cells expressing POT1-WT, POT1-ΔOB, and POT1-K90E were transduced with a catalytically inactive Cas9 fused to the KRAB domain (dCas9-KRAB) and a hCRISPRi v2.1 sgRNA library, and were passaged at ∼1000× coverage for approximately nine population doublings (PD). We performed next-generation sequencing (NGS) and determined the relative change in sgRNA abundance between PD = 9 and PD = 0 (Fig. 1A). Normalized sgRNA counts from two independent replicates were analyzed using the Bayesian analysis of gene essentiality (BAGEL) algorithm to determine “essential” genes (Hart et al. 2015; Hart and Moffat 2016). The likelihood of a gene depletion causing a fitness defect is represented as a Bayes factor (BF) score that is generated using empirically determined reference gene sets (Hart et al. 2017). Using a threshold of BF ≥3 as a cutoff (Hart et al. 2017; Lenoir et al. 2018) we identified 1483 “essential” genes in cells expressing POT1-WT, whereas cells expressing POT1-K90E and POT1-ΔOB yielded a list of 1721 and 1532 “essential” genes, respectively (Supplemental Table S1). Precision-recall curves confirmed the robustness of the CRISPRi screens (Supplemental Fig. S1C). Furthermore, we compared the BF scores from two independent screens and observed a strong correlation (r^2^ > 0.7 [Supplemental Fig. S1D] and Pearson's coefficient > 0.85 [Supplemental Table S2]). To gain a better understanding of the genetic interactions with *POT1* mutations in the context of untransformed cells, we repeated the CRISPRi screen in p53-deficient retinal pigmental epithelial cells immortalized with hTERT (RPE-1). We transduced RPE-1 p53^−/−^ cells expressing POT1-WT, POT1-K90E, and POT1-ΔOB with dCas9-KRAB (Supplemental Fig. S1B) and a hCRISPRi v2.1 sgRNA library. Cells were passaged for 14 PDs; we then performed NGS and applied BAGEL analysis to determine the list of “essential” genes in the different genetic conditions (Fig. 1A; Supplemental Figs. S1E,F; Supplemental Table S1).

### Biological processes related to DNA replication, mitosis, and the nuclear pore complex display a genetic interaction with mutant POT1

Having confirmed the robustness of the CRISPRi screens in two independent cell lines (Supplemental Fig. S1C), we then determined the genes that differentially impact the growth of cells expressing mutant POT1. “Essential” genes identified in cells expressing POT1-K90E and POT1-ΔOB were assigned a *z*-score that we calculated from the difference in BF scores between mutant and wild-type POT1. Genes with a *z*-score ≥ 0.53 were considered synthetic lethal (SL) candidates (Fig. 1B,C; Supplemental Table S1). The majority of candidate SL genes in RPE-1 p53^−/−^ cells expressing POT1-K90E and POT1-ΔOB were classified as “nonessential” in cells expressing POT1-WT (Supplemental Fig. S1F; Supplemental Table S1). Furthermore, we noted a significant overlap in SL genes between POT1-K90E- and POT1-ΔOB-expressing cells (Supplemental Fig. S2A). Notably, *POT1* appeared as an SL hit in cells expressing POT1-ΔOB, and to a lesser extent, POT1-K90E (Fig. 1B,C). This provided internal validation for our screen since a reduction in endogenous levels of POT1 with CRISPRi is expected to exacerbate telomere dysfunction caused by mutant POT1. A common SL hit that appeared in three of the four screens was *HUS1,* a member of the 9-1-1 complex that activates ATR signaling in response to replication stress (Saldivar et al. 2017). We transduced RPE-1 p53^−/−^ cells expressing dCas9-KRAB and the various POT1 alleles with sgRNAs against *HUS1* and determined cell viability using CellTiter-Glo (Supplemental Fig. S2B). In an independent approach, we used shRNA-mediated depletion of HUS1 and monitored cell proliferation using the Incucyte S3 live-cell analysis system, which uses live-cell imaging to monitor cell proliferation in real time (Supplemental Fig. S2C). Our data corroborated the results of the CRISPRi screen, whereby HUS1 depletion conferred a greater fitness defect in cells expressing mutant POT1 (Supplemental Fig. S2B,C).

To identify the cellular processes that are essential for the growth of cells expressing *POT1* OB-fold mutations we used pathway enrichment analysis. We analyzed the SL gene lists from the genome-wide screens using the PANTHER tool (Mi et al. 2019) and identified pathways that were common to cells expressing POT1-K90E and POT1-ΔOB. Overrepresentation analysis revealed an enrichment of several REACTOME pathways, including “G2/M transition,” “homology directed repair,” “SUMOylation of DNA repair,” “DNA replication,” and “resolution of sister chromatid cohesion” (Fig. 1D; Supplemental Table S3). These pathways are broadly categorized as processes that mediate the cellular response to DNA replication stress, and in agreement with our previous report, firmly establish the impact of POT1 alterations on telomere replication (Pinzaru et al. 2016). In addition, our screen identified the “NPC disassembly” pathway as essential for growth of cells with *POT1* OB-fold mutations (Fig. 1D; Supplemental Table S3). Several nucleoporins (NUPs) displayed SL interactions with POT1-K90E and POT1-ΔOB, including subunits of the Y complex (NUP37, NUP43, NUP85, NUP107, NUP160, and SEC13) and the nuclear pore basket (NUP153 and TPR) (Fig. 1B,C).

### A proteomic approach uncovers the telomere interactome in cells expressing mutant POT1

**Figure 2.**
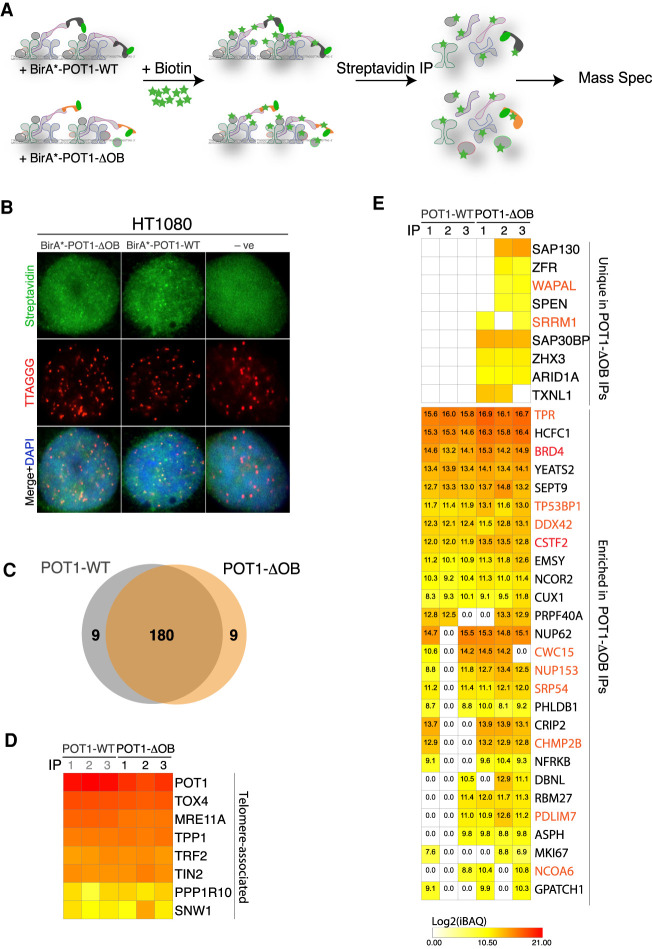
Proteomic analysis of the telomere interactome in cells expressing a *POT1* OB-fold mutation. (*A*) Schematic of the biotin ligase (BirA*) purification scheme to characterize the telomere proteome in cells expressing BirA*-POT1-WT and BirA*-POT1-ΔOB. BirA* covalently attaches biotin to lysine residues on vicinal proteins. Labeled proteins are recovered by streptavidin immunoprecipitation (IP) and identified using liquid chromatography–mass spectrometry. IPs were performed in triplicate for each POT1 variant. (*B*) Indirect immunofluorescence (IF) for streptavidin (green) coupled with fluorescence in situ hybridization for telomeres (FISH) (red) in HT0180 cells with the indicated treatment and following overnight incubation with excess biotin. Cells expressing an empty vector were used as negative control for the staining. (*C*) Venn diagram of the overlap between POT1-WT and POT1-ΔOB hits identified in two or more IPs. (*D*) Heat map of the log_2_ transformed intensity-based absolute quantification (iBAQ) values for telomere-associated proteins recovered in cells expressing BirA*-POT1-WT or BirA*-POT1-ΔOB. *n* = 3 independent IPs. (*E*) Heat map of the log_2_ transformed iBAQ values of proteins enriched at telomeres in POT1-ΔOB cells. (*Top*) Proteins uniquely present in two or more IPs in POT1-ΔOB-expressing cells. (*Bottom*) iBAQ values for proteins with ≥50% enrichment (≥0.6 in log_2_) in two or more IPs in cells expressing POT1-ΔOB compared with those expressing POT1-WT. Values were compared in a paired manner (i.e., IP#1 for POT1-WT with IP#1 for POT1-ΔOB). Highlighted in red are enriched proteins that have been identified as SL candidates in the genome-wide screen (Supplemental Table S1).

To complement our genetic screen, we used a proteomic approach that interrogated the changes in telomere chromatin caused by dysfunctional POT1. To do so, we applied the proximity-labeling method—termed BioID—that harnesses a promiscuous *E. coli* biotin ligase (BirA*) capable of covalently attaching biotin on proximal proteins (Roux et al. 2012). Myc-tagged BirA* was fused to POT1-WT and POT1-ΔOB and stably expressed in HT1080 cells (Fig. 2A). As a control, we showed that cells expressing BirA*-POT1-ΔOB displayed increased levels of telomere dysfunction-induced foci (TIFs), determined by the accumulation of DNA damage factor 53BP1 at telomeres (Supplemental Fig. S3A; Takai et al. 2003). Furthermore, immunofluorescence (IF) confirmed that upon treatment of cells with biotin, the fusion proteins stimulated the biotinylation of factors in the vicinity of telomeres (Fig. 2B). We then performed streptavidin immunoprecipitation (IP) on lysates from cells expressing the various POT1 alleles (Supplemental Fig. S3B). Unique peptides were identified using liquid chromatography-mass spectrometry (LC-MS) and their abundance determined using intensity-based absolute quantification (iBAQ) (Supplemental Table S4; Schwanhäusser et al. 2011). We excluded proteins common to a contaminant repository for affinity purification (CRAPome) (Mellacheruvu et al. 2013) and ones recovered in less than two IPs. Based on these criteria, we identified ∼200 proteins that were enriched at telomeres regardless whether they were bound by POT1-WT or POT1-ΔOB (Fig. 2C–E; Supplemental Fig. S3C; Supplemental Table S4). Common hits included members of the shelterin complex—TPP1, TIN2, and TRF2—that were recovered with similar abundance, and other previously identified telomeres-associated proteins (Fig. 2D; Déjardin and Kingston 2009; Grolimund et al. 2013; Garcia-Exposito et al. 2016). We also identified 36 proteins that were unique to or substantially enriched (>50% in at least two IPs) at telomeres in the presence of POT1-ΔOB (Fig. 2E). These included 53BP1 and its interacting partners, SAP130, ARID1A, and ZFR (Fig. 2E; Gupta et al. 2018). In addition, we retrieved 13 factors that were also identified as SL candidates in the CRISPRi screen, including the NPC subunits, TPR and NUP153 (Figs. 1C, 2E; Supplemental Tables S1, S4).

### Activation of mitotic DNA synthesis (MiDAS) in response to POT1 dysfunction

**Figure 3.**
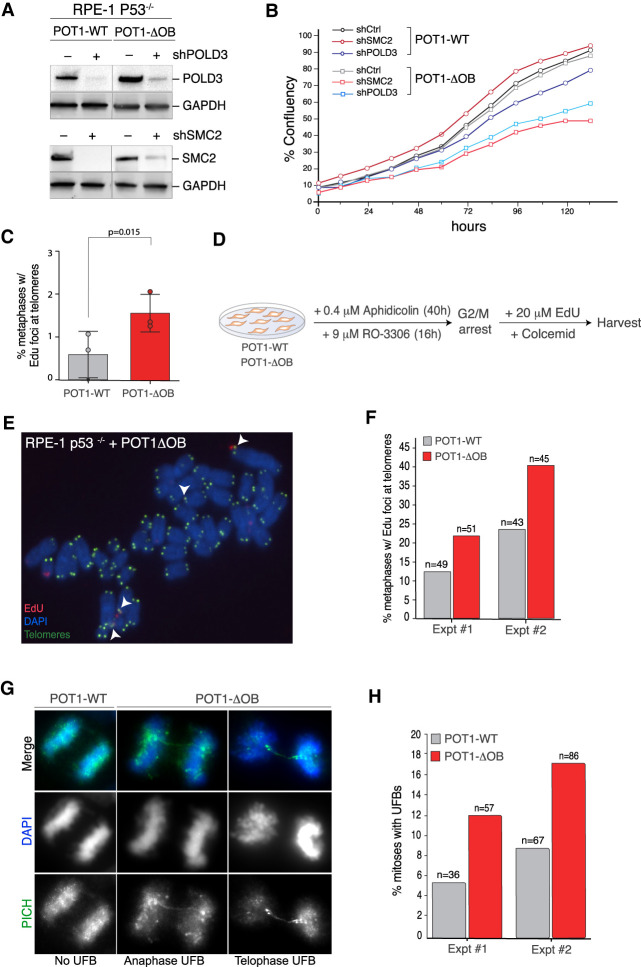
POT1 dysfunction triggers mitotic DNA synthesis (MiDAS) at telomeres. (*A*,*B*) Validation of growth defects upon inhibition of MiDAS factors, SMC2 and POLD3, in cells expressing mutant POT1. (*A*) Western blot analysis for POLD3 and SMC2 in cells with the indicated treatment. (*B*) Graph depicting cellular growth monitored in real time by Incucyte in the indicated cells. (*C*) Analysis of MiDAS in asynchronous HT1080 cells expressing POT1-WT and POT1-ΔOB. Quantification of the number of metaphases with EdU foci at telomeres from asynchronous cells. Mean ± SD of three independent experiments (*n* = 634 metaphases for POT1-WT and 597 for POT1-ΔOB): Student's *t*-test, paired, and one-tailed. (*D*) Setup of MiDAS experiment in RPE-1 p53^−/−^ cells expressing POT1-WT and POT1-ΔOB. Cells were treated with aphidicolin (0.4 µM) for 40 h and with Cdk1 inhibitor RO-3306 (9 µM) during the last 16h of the aphidicolin treatment. Following release from the G2/M block, cells were incubated with 20 µM EdU and Colcemid for 50–60 min, before metaphases were harvested. (*E*,*F*) Analysis of mitotic DNA synthesis (MiDAS) in RPE-1 p53^−/−^ cells exogenously expressing POT1-WT and POT1-ΔOB following the treatment as in *D*. (*E*) Representative image of EdU incorporation at telomeres; arrowheads point to EdU foci at telomeres. (*F*) Quantification of the number of metaphases with EdU incorporation at telomeres; two independent experiments. (*G*,*H*) Analysis of ultrafine DNA bridges (UFBs) in U2OS cells exogenously expressing POT1-WT and POT1-ΔOB. (*G*) Representative images of mitotic UFBs detected by indirect immunofluorescence for PICH (green); DNA is stained with DAPI in blue. (*H*) Quantification of PICH UFBs in two independent experiments (total *n* > 100 mitoses for each condition).

When faced with incomplete DNA replication in the S/G2 phases, mammalian cells use a salvage pathway termed MiDAS that completes copying of underreplicated regions in the early stages of mitosis (Minocherhomji et al. 2015; Özer and Hickson 2018). Several MiDAS factors, including WAPAL, SMC2, and POLD3 (Minocherhomji et al. 2015) appeared as SL hits in cells expressing mutant POT1 (Fig. 1B,C) and depletion of SMC2, POLD3, and WAPAL impaired the proliferation of cells expressing POT1-ΔOB compared with cells expressing wild-type POT1 (Fig. 3A,B; Supplemental Fig. S4A,B). WAPAL was also enriched at telomeres in cells expressing POT1-ΔOB (Fig. 2E). To test whether mutations in *POT1* trigger MiDAS at telomeric loci, we monitored the incorporation of base thymidine analogue EdU during mitosis (Garribba et al. 2018). In asynchronous cells, we detected an approximate twofold increase in the incorporation of mitotic EdU in cells expressing POT1-ΔOB compared with POT1-WT (Fig. 3C; Supplemental Fig. S4C). It has been previously noted that MiDAS at telomeres is a rare event in telomerase positive cells (Min et al. 2017; Özer et al. 2018), we therefore enriched for MiDAS events by treating RPE-1 p53^−/−^ cells with low doses of aphidicolin and the Cdk1 inhibitor RO-3306 (Fig. 3D; Supplemental Fig. S4D) as previously described (Özer et al. 2018). Our results further confirmed a significant increase in MiDAS in the presence of POT1-ΔOB (Fig. 3E,F; Supplemental Fig. S4E).

A characteristic feature of replication defects carried into mitosis is the appearance of ultra-fine anaphase bridges (UFBs), which arise when a cell enters anaphase without having resolved replication intermediates. UFBs manifest as thin DNA entanglements typically coated with PICH (Plk1 interaction checkpoint helicase) and are observed at fragile sites (Chan et al. 2009), including telomeres (Barefield and Karlseder 2012). We examined POT1 mutant cells in mitosis and noticed an enrichment in PICH-labeled bridges compared with control cells (Fig. 3G,H). Notably, we did not detect an increase in the frequency of chromatin bridges and lagging chromosomes upon POT1 dysfunction (Supplemental Fig. S4F,G). Furthermore, we observed no difference in anaphase onset (Supplemental Fig. S4H,I), indicating that replication stress in response to POT1 alterations did not lead to overt defects in mitotic progression and chromosome segregation. Based on these results, we conclude that increased MiDAS enables POT1 mutant cells to cope with telomere replication defects, and that unresolved replication intermediates that progress through mitosis give rise to UFBs.

### The NPC is necessary for maintaining telomere stability in cells with mutant POT1

**Figure 4.**
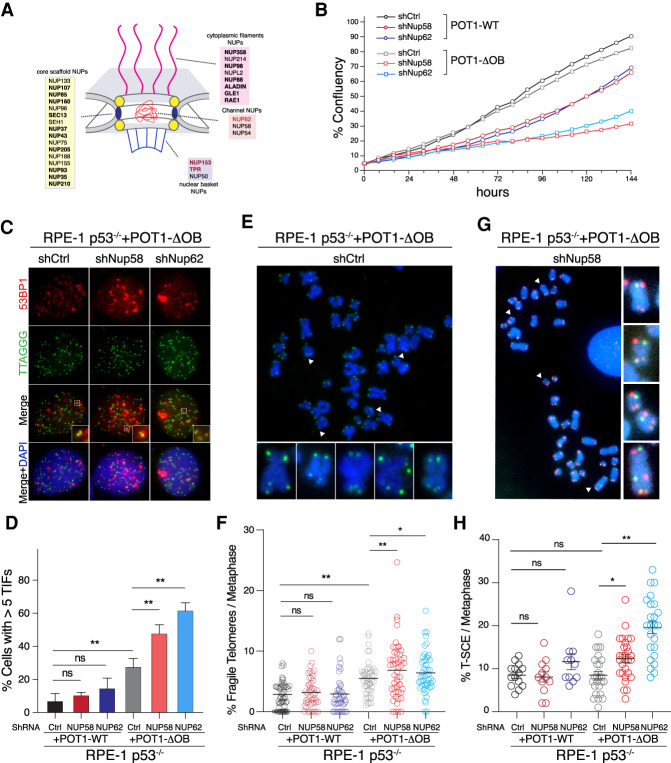
The NUP62 subcomplex is necessary to maintain telomere integrity in cells carrying mutant POT1. (*A*) Schematic of the nuclear pore complex in mammalian cells (Kabachinski and Schwartz 2015). Highlighted in bold are SL hits with POT1 mutants and in red are nucleoporins (NUPs) enriched in the POT1-ΔOB BioID IPs. NUP153 and TPR were hits in both screens. (*B*) Incucyte growth analysis of RPE-1 p53^−/−^ cells expressing POT1-WT and POT1-ΔOB, treated with shRNAs against NUP62, NUP58, and scramble control. Cell proliferation was monitored over 160h. Graph representing data from two independent experiments. (*C*) Representative images displaying telomere dysfunction-induced foci (TIFs) in RPE-1 p53^−/−^ cells expressing POT1-ΔOB and treated with shRNAs against NUP58 and NUP62, as well as control shRNA. 53BP1 in red is detected by indirect immunofluorescence, and telomeres are marked with FISH in green. DNA is counterstained with DAPI in blue. (*D*) Quantification of the percentage of cells with five or more TIFs in RPE-1 p53^−/−^ cells with the indicated treatment. Graph represents the mean of *n* = 3 independent experiments with SD (two-tailed *t*-test). (*E*,*F*) Analysis of telomere fragility in RPE-1 p53^−/−^ cells expressing POT1-WT and POT1-ΔOB and treated with the indicated shRNA. (*E*) Representative images showing metaphase chromosomes from POT1-ΔOB cells with fragile telomeres. Arrowheads indicate examples of fragile telomeres on the metaphase. Telomeres were stained with FISH in green, while DNA is detected with DAPI in blue. (*F*) Quantification of fragile telomeres per metaphase in cells with the indicated treatments. Graph represents mean of four independent experiments (*n* > 40 metaphase per condition) with SD (one-way ANOVA test). (*G*,*H*) Telomere sister chromatid exchange (T-SCE) analysis in cells with the indicated treatment. (*G*) Image depicting metaphase chromosomes with T-SCE events (white arrows). Telomeres were stained with FISH in green and red and DNA is counterstained with DAPI in blue. (*H*) Quantification of T-SCE events per metaphase in the indicated cells (*n* > 15 metaphases per condition).

The CRISPRi screen highlighted a genetic interaction between *POT1* OB-fold mutations and several nucleoporins (NUPs) that belong to different nuclear pore subcomplexes (Fig. 4A). In addition, TPR, NUP62, and NUP153 were enriched in the vicinity of telomeres bound by POT1-ΔOB (Fig. 2E). To uncover the function of the NPC during POT1 dysfunction, we treated RPE-1 p53^−/−^ cells expressing POT1-ΔOB and POT1-WT with shRNAs against several NPC subunits, including the NUP62 subcomplex members, NUP62 and NUP58, as well as the basket NUPs, TPR and NUP153 (Supplemental Fig. S5A,B). We monitored cell proliferation and found that the depletion of the NUP62 and NUP58 subunits preferentially impaired the growth of POT1-ΔOB cells relative to cells expressing POT1-WT (Fig. 4B). Consistent with previous reports (Hockemeyer et al. 2007), expression of POT1-ΔOB induced a telomere DNA damage response that was exacerbated upon the depletion of NUP62, NUP58, TPR, and NUP153 (Fig. 4C,D; Supplemental Fig. S5C,D). In contrast, depletion of these NUPs in cells expressing POT1-WT did not elicit a TIF response (Fig. 4C,D; Supplemental Fig. S5C,D). We next examined the impact of NUP depletion on telomere fragility, which is an established marker of telomere replication stress (Martinez et al. 2009; Sfeir et al. 2009). Chromosome analysis revealed that inhibition of NUPs increased the incidence of fragile telomeres in cells expressing POT1-ΔOB cells (Fig. 4E,F; Supplemental Fig. S5E), suggestive of a role for an intact NPC in resolving telomeric replication stress caused by POT1 dysfunction. To corroborate these results, we used CRISPR/Cas9 to knock-in two cancer-associated mutations at the endogenous *POT1* locus. Specifically, we generated RPE-1 cells harboring POT1-K90E and POT1-F62V, which disrupts POT1 binding to ssDNA (Supplemental Fig. S6A; Pinzaru et al. 2016). We then depleted NUP62 and NUP58 (Supplemental Fig. S6B) and noted an increase in telomere dysfunction and fragility relative to cells expressing a scramble shRNA control (Supplemental Fig. S6C–E). To rule out that NUP depletion triggers telomere dysfunction indirectly by compromising nuclear import, we performed cellular fractionation experiments following the depletion of NUP62 and NUP58, and showed efficient nuclear accumulation of shelterin subunits and other proteins involved in the DNA damage response (Supplemental Fig. S5F).

In budding yeast, stalled replication forks relocate to the nuclear pore in order to promote fork restart and prevent CAG repeat instability through recombination (Su et al. 2015). To test whether the NPC prevents deleterious telomere–telomere recombination at stalled replication forks, we measured the frequency of telomere sister chromatid exchange (T-SCE) events following the depletion of two NUP subunits in cells expressing POT1-WT and POT1-ΔOB. We show that inhibition of the NUP62 subcomplex significantly increased the frequency of T-SCEs in POT1 mutant cells (Fig. 4G,H). Cumulatively, our data highlight a direct role for the NPC in maintaining telomere stability in response to replication stress in the context of mutant POT1 by preventing unwanted recombination between telomere repeats.

### POT1 dysfunction promotes F-actin-dependent telomere repositioning to the nuclear periphery

**Figure 5.**
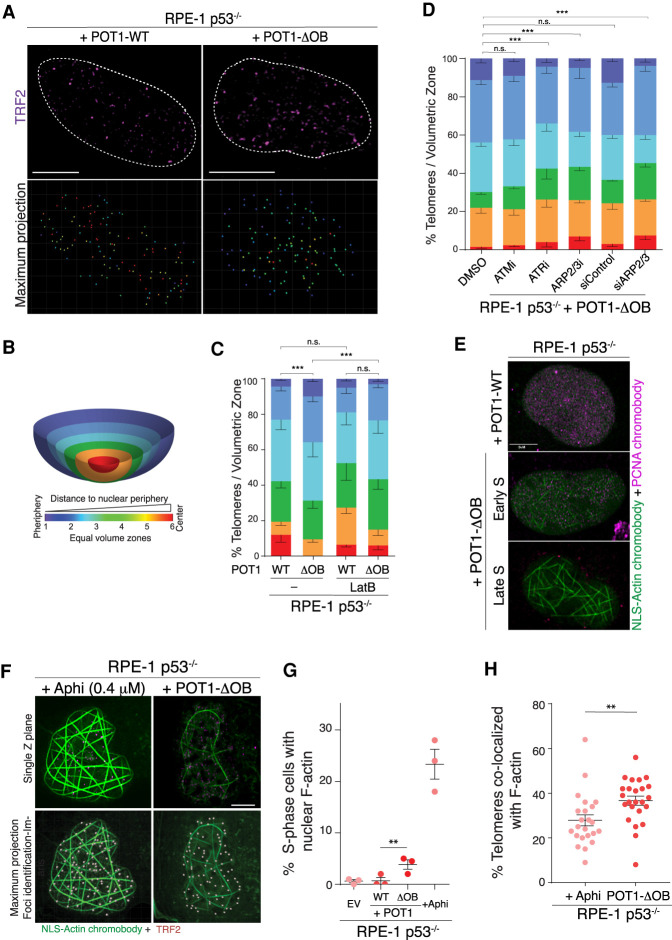
Nuclear F-actin polymerization in POT1-ΔOB cells facilitates the relocalization of telomeres to the nuclear periphery. (*A*) Representative superresolution microscopy of a three-dimensional (3D) image through the nuclear volume of fixed RPE-1 p53^−/−^ cells expressing POT1-WT and POT1-ΔOB. S-phase cells were marked with PCNA (not shown) and telomeres detected with an anti-TRF2 antibody in magenta. The *top* image is a single z-plane through the nuclear center. The *bottom* image is a maximum projection rendering of all telomeres with their distance from the nuclear edge color coded. (*B*) Telomeres were identified throughout the nuclear volume and their distance to the nuclear periphery calculated using the Imaris 8.4.1 software. DAPI was used to segment nuclei into six equal volume zones from nuclear center to the periphery and each telomere assigned to the corresponding zone. The zone for each telomere relative to the nuclear periphery is identified via color coding. (*C*) Quantification of telomere localization in the nucleus of cells imaged in *A*, in the absence or presence of 0.2 µM Latrunculin B (LatB) treatment (24 h prior to fixation). Graph represents distribution of telomeres with respect to the nuclear periphery. Mean ± SEM (χ^2^ test). *n* ≥ 1805 telomeres from >19 nuclei and three independent experiments. (*D*) Quantification of telomere localization in the nucleus of cells with the indicated treatment. Graph represents distribution of telomeres with respect to the nuclear periphery. Mean ± SEM (χ^2^ test). *n* ≥ 6004 telomeres from >63 nuclei and three independent experiments. (*E*) Representative superresolution microscopy of single Z-planes taken from 3D images through the nuclear volume of fixed RPE-1 p53^−/−^ cells expressing POT1-WT and POT1-ΔOB. Cells were transfected with NLS-GFP-Actin and Tag-RFP-PCNA chromobodies 48 h prior to fixation. (*F*) Representative superresolution microscopy of 3D images through the nuclear volume of fixed parental RPE-1 p53^−/−^ cells and cells expressing POT1-ΔOB. Cells were transfected with an NLS-GFP-Actin chromobody 48 h prior to fixation and telomeres were detected with an anti-TRF2 antibody. Parental cells were treated with 0.4 µM aphidicolin for 24 h prior to fixation. (*G*) Quantification of filamentous-actin (F-actin) positive S-phase nuclei from the images depicted in *E*. Each data point represents an individual biological replicate. *n* = 3 independent experiments with >168 nuclei analyzed per experiment. SEM with Fisher's exact test. (*H*) Quantification of the percentage of telomeres that colocalized with nuclear F-actin in experiments highlighted in *G*. *n* = 3 independent experiments with >23 nuclei and >1656 telomeres. SEM with Mann–Whitney test. (**) *P* < 0.001; (***) *P* < 0.0005. Scale bar, 5 µm.

Our results implicate the NPC in the resolution of replication-related telomere defects in human cells harboring *POT1* mutations. This raised the obvious question of whether telomeres in POT1 mutant cells are targeted to the nuclear periphery where nucleoporins reside. To investigate potential telomere targeting to the nuclear edge, we visualized telomeres within the three-dimensional nuclear volume. RPE-1 p53^−/−^ cells expressing POT1-ΔOB and POT1-WT were stained with an anti-TRF2 antibody to label the telomeres and PCNA to mark S-phase cells (Fig. 5A). Replicating cells were visualized using Airyscan superresolution microscopy. We marked the nuclear surface area with DAPI and segmented the nucleus into six equal volume zones from the nuclear center to the nuclear edge (Fig. 5B). Telomeres were assigned to their respective volumetric zone depending on their three-dimensional localization (Fig. 5A,B; Supplemental Fig. S7A,B). We observed that telomeres in POT1-ΔOB expressing cells were enriched towards the nuclear edge in contrast to cells expressing POT1-WT (Fig. 5B,C). As a control, we inhibited the shelterin subunit TRF2 that protects telomeres from ATM signaling and nonhomologous end-joining (Supplemental Fig. S8A,B; Cesare et al. 2013). Analysis of telomere localization using superresolution microscopy indicated that cells treated with TRF2 shRNA did not display telomere relocalization to the nuclear periphery (Supplemental Fig. S8A,B). Consistent with replication stress underlying telomere repositioning in POT1-mutant cells, we observed a significant reduction in telomere mobilization to the nuclear periphery upon inhibition of ATR but not ATM kinase (Fig. 5D).

A novel mechanism that mediates DNA relocalization emerged from recent literature, where polymerization of nuclear F-actin filaments was shown to facilitate the mobilization of damaged DNA toward the nuclear periphery. This includes the directed movement of heterochromatic DSBs in *D. melanogaster* and stalled replication forks in human cells (Ryu et al. 2015; Caridi et al. 2018; Lamm et al. 2018; Schrank et al. 2018). We therefore monitored cell cycle-dependent nuclear F-actin polymerization upon telomere dysfunction. Cell-expressing POT1 variants were labeled with an NLS-GFP-actin chromobody that detects nuclear F-actin filaments and costained with an RFP-PCNA chromobody to mark cells in S-phase (Fig. 5E; Supplemental Fig. S8C). As a control, we showed that treatment of wild-type cells with low levels of aphidicolin (0.4 µM), which causes global replication stress, triggered significant nuclear F-actin filament formation (Fig. 5F,G). Furthermore, we noted an enrichment of S-phase cells displaying nuclear F-actin in cells expressing POT1-ΔOB compared with POT1-WT (Fig. 5E–G). Notably, the induction of nuclear F-actin in the presence of POT1-ΔOB was less robust than with aphidicolin treatment. Telomeric DNA represents a small fraction of the entire genome, which likely explains why telomere-specific replication stress results in a milder effect on nuclear F-actin polymerization than genome-wide replication stress induced by aphidicolin. Importantly, the colocalization of telomeres with nuclear F-actin filaments was significantly enriched in cells expressing POT1-ΔOB relative to parental cells treated with aphidicolin (Fig. 5H). It is worth noting that the colocalization of telomeres with F-actin in cells expressing POT1-WT could not be analyzed due to the paucity of S-phase cells with nuclear actin filaments.

The ARP2/3 actin nucleating complex was shown to promote F-actin-dependent mobility of stressed replication forks (N Lamm and AJ Cesare, unpubl.). To test whether actin polymerization promoted telomere mobility in POT1-ΔOB expressing cells, we independently inhibited F-actin polymerization with Latrunculin B (LatB), ARP2/3 chemical inhibition, and APR2/3 siRNA. In all cases, inhibition of actin polymerization conferred a significant reduction in the peripheral localization of telomeres in POT1-ΔOB expressing cells (Fig. 5C,D; Supplemental Fig. S8D,E). Furthermore, POT1-ΔOB-expressing cells treated with LatB displayed increased telomere dysfunction and fragility (Supplemental Fig. S9). In conclusion, our data indicate that actin-dependent forces reposition telomeres to the nuclear periphery in response to replication stress caused by POT1 dysfunction.

## Discussion

### Replication stress as a major vulnerability in cells with mutant POT1 alleles

Sequencing of cancer genomes identified *POT1* mutations in several solid tumors and hematological malignancies (Quesada et al. 2012; Lazzerini-Denchi and Sfeir 2016; Speedy et al. 2016; McMaster et al. 2018). To better understand the basis by which mutant *POT1* alleles foster tumorigenesis, we performed a genome-wide CRISPRi screen that identified a set of pathways essential for the survival of cells with *POT1* OB-fold mutations. These pathways included DNA replication, S phase, homology directed repair, SUMOylation of DNA replication proteins, and nuclear pore complex disassembly (Fig. 1D), which collectively underscore replication stress as a major defect associated with *POT1* mutations. While nuclease-based CRISPR screens often yield stronger SL hits, a CRISPR interference-based approach has an advantage as it uncovers genetic interactions that involve essential genes. Indeed, the identification of SL interactions between mutant POT1 and subunits of the NPC, which are essential for cellular survival (Kabachinski and Schwartz 2015), were possible because of the incomplete repression of genes by CRISPRi (Gilbert et al. 2014).

We complemented our genetic screen with a biotin-dependent labeling approach (Roux et al. 2012) and identified factors that are in proximity to telomeres bound by mutant POT1. Notable telomere interactors that were enriched in POT1-ΔOB expressing cells that also appeared as SL candidates in the CRISPRi screen included WAPAL and NPC subunits (Fig. 2). Consistent with POT1 dysfunction leading to incomplete telomere replication in S phase, we detected an increase in MiDAS in cells expressing POT1-ΔOB (Fig. 3). Furthermore, inhibition of MiDAS factors, SMC2, POLD3, and WAPAL compromised growth of cells expressing mutant POT1 (Fig. 3; Supplemental Fig. S4A,B). While we cannot rule out that the observed synthetic lethality is due to additional functions of these factors, our results suggest that MiDAS acts as a salvage pathway to complete synthesis of partially replicated telomere in mitosis. Our study also highlights components of MiDAS as potential targets for the treatment of an increasing number of tumors associated with *POT1* mutations.

### A conserved function for the NPC in resolving damage in repetitive DNA

The link between the nuclear pore complex and DNA repair was first established in yeast based on the observation that mutations in several NUPs cause hypersensitivity to DNA damage agents and are lethal with mutations that impair HR (Bennett et al. 2001; Loeillet et al. 2005; Therizols et al. 2006). Since then, increasing evidence demonstrated the relocalization of persistent DNA breaks to the vicinity of nuclear pores in *S. cerevisiae*. Such lesions included collapsed replication forks, HO-induced breaks, eroded telomeres, and CAG repeats (Nagai et al. 2008; Khadaroo et al. 2009; Oza et al. 2009; Su et al. 2015; Churikov et al. 2016). In all cases, relocalization to the nuclear pore was necessary for efficient DNA repair and to prevent genome instability. Transient tethering of damaged DNA to the nuclear pore was also noted in *Drosophila melanogaster* cells, particularly at breaks incurred within heterochromatic loci (Ryu et al. 2015). With regard to mammalian cells, no direct role for the NPC in repairing DNA lesions has been reported thus far. Here, we provide the first genetic evidence that links the NPC with maintenance of repetitive DNA and demonstrate targeting of damaged telomeres to the nuclear periphery.

Mouse telomeres rendered dysfunctional as a result of TRF2 deletion displayed increased mobility but did not cluster at the nuclear periphery (Lottersberger et al. 2015). Similarly, FokI-induced DSBs at telomeres in U2OS cells triggered telomere clustering without apparent relocalization to the periphery (Cho et al. 2014). In contrast, our data suggest that telomeres undergoing replication stress have distinct properties that promote their targeting to the nuclear edge (Fig. 5). Telomere uncapping upon TRF2 loss and FokI induced telomere breaks trigger ATM kinase activation akin to the canonical DSB response (Celli and de Lange 2005; Doksani and de Lange 2016). In contrast, stalled forks as a result of *POT1* inhibition activate the ATR kinase pathway (Pinzaru et al. 2016). We show here that ATR facilitates telomere repositioning to the nuclear periphery, and that telomere interaction with NPCs prevents unwanted exchange of telomere sequence between sister chromatids (Figs. 4G,H, 5D). It is possible that telomere targeting to the periphery is driven by a specific type of substrate that is associated with stalled replication, such as a reversed or collapsed fork.

### Nuclear F-actin filaments facilitate telomere repositioning to the nuclear periphery

Repositioning of DNA breaks away from their primary site is common to several genomic loci and has been observed in multiple organisms (Ryu et al. 2015; Caridi et al. 2018; Smith et al. 2018; Marnef et al. 2019). The mechanistic understanding of damage-induced DNA mobility has only begun to emerge. Genetic studies in yeast and flies identified a critical role for SUMOylation, mainly by the Slx5–Slx8 SUMO-targeted ubiquitin ligase (STUbL), in the spatial and temporal regulation of break repair (Nagai et al. 2008; Ryu et al. 2015; Su et al. 2015; Churikov et al. 2016; Horigome et al. 2016). Interestingly, “SUMOylation of DNA damage response and repair proteins” and “SUMOylation of DNA replication proteins” appeared as pathways that are synthetic lethal with mutant POT1 (Fig. 1D) and the Slx5/8 human ortholog *RNF4* showed a genetic interaction with POT1-ΔOB (Supplemental Table S1). While RNF4 has been previously linked to DSB repair at telomeres in the context of TRF2 loss (Groocock et al. 2014), its function in response to telomere replication defects remains to be investigated.

Recent reports implicated nuclear F-actin filament formation during the directional mobility of damaged DNA in *D. melanogaster* as well as human cells (Caridi et al. 2018; Lamm et al. 2018; Schrank et al. 2018). Our data are consistent with nuclear F-actin polymerization underlying the process of telomere relocalization to the periphery. Specifically, we observed F-actin filament formation in S-phase cells expressing mutant POT1 (Fig. 5E–H) and show that blocking actin polymerization with LatB reduced the fraction of telomeres at the nuclear periphery (Fig. 5C,D) and promoted increased telomere dysfunction and fragility (Supplemental Fig. S9). Based on these data, we propose a model where actin polymerization potentially facilitates the targeting of replication-defective telomeres to the NPC. These relocalization events are expected to isolate stalled forks involving telomeric repeats, presumably to prevent illegitimate recombinational repair and facilitate fork restart (Freudenreich and Su 2016). In summary, our study has uncovered a conserved mechanism from yeast to humans that targets persistent damage at specialized loci to the nuclear periphery to ensure genome stability.

## Materials and methods

### Cell culture and treatments

The p53-null, hTERT immortalized retinal pigment epithelial cell line, RPE-1 *TP53^−/−^*, was a gift from the Meng-Fu Bryan Tsou Laboratory at Memorial Sloan Kettering Cancer Center. RPE-1 *TP53^−/−^*, RPE-1 and osteosarcoma U2OS cells were cultured in DMEM supplemented with 10% FBS (Gibco), 2 mM L-glutamine (Sigma), 100 U/mL penicillin (Sigma), 0.1 μg/mL streptomycin (Sigma), and 0.1 mM nonessential amino acids (Invitrogen). HT1080 fibrosarcoma cells were grown in DMEM supplemented with 10% BCS (bovine calf serum; Hyclone), 2 mM L-glutamine (Sigma), 100 U/mL penicillin (Sigma), 0.1 μg/mL streptomycin (Sigma) and 0.1 mM nonessential amino acids (Invitrogen). The cells were tested for mycoplasma using the LookOut mycoplasma PCR detection kit (Sigma MP0035), following the manufacturer's instructions and using the JumpStart Taq DNA polymerase (Sigma D9307). For the latrunculin B treatment experiments, the cells were treated with 0.2 µM Latrunculin B for 24 h or with dimethyl sulfoxide (DMSO), prior to collection for analysis. For the kinase and ARP2/3 inhibitor experiments, the cells were treated DMSO as controls, or with 1 µM ATM inhibitor KU-55933, 1 µM ATR inhibitor VE-822 (Selleckchem S7102), and with 200 µM ARP2/3 inhibitor CK666.

### Plasmids

Human POT1 variants (wild-type POT1, POT1-ΔOB, and POT1-K90E) were subcloned into the lentiviral construct pHAGE2-EF1a-MCS-IRES-blast and pWZL-N-Myc-IRES-hygro from pLPC-N-Myc-POT1. The pHAGE2-EF1a-MCS-IRES backbone was a gift from the Matthias Stadtfeld laboratory at New York University Langone Health. Wild-type POT1 and POT1-ΔOB were also subcloned downstream from Myc-FLAG-BirA* to generate pLPC-N-Myc-FLAG-BirA*-POT1/POT1-ΔOB constructs.

The hCRIPSRi-v2 library top five sgRNAs/gene (Addgene 83969) (Horlbeck et al. 2016), was a gift from the Jonathan Weissman laboratory at University of California at San Francisco. The lentiviral construct expressing dCas9-HA-NLS-TagBFP-Krab-NLS was a gift from the Jonathan Weissman laboratory. We generated a dCas9-HA-NLS-mCherry-Krab-NLS construct by replacing the TagBFP with mCherry, using BamHI and NdeI sites added on mCherry during PCR amplification with the Q5 high-fidelity DNA olymerase (NEB M0491). The pBabe-H2B-GFP plasmid (Addgene 26790) was a gift from the Susan Smith lab at NYU Langone Health. All shRNA experiments were performed using the pLKO.1-puro lentiviral vector backbone. The constructs were transduced into human cells using established transduction protocols for generating stable cell lines.

### CRISPRi screen: infection, cell culture, library preparation

The screen was performed with the hCRIPSRi-v2 library (Horlbeck et al. 2016), which comprises five optimized sgRNAs per gene, for a total of 102640 sgRNAs targeting close to 19,000 genes. The library also includes ∼1900 nontargeting control sgRNAs. The hCRIPSRi-v2 is a two-vector system, where the dCas9-KRAB fusion is expressed separately from the sgRNA library. The screen was performed in duplicate in HT1080 cells expressing dCas9-HA-NLS-BFP-Krab-NLS and pWZL-Myc-POT1-IRES-Hygro, pWZL-Myc-POT1-K90E-IRES-Hygro or pWZL-Myc-ΔOB-POT1-IRES-Hygro. The whole-genome screen was repeated once in RPE-1 *TP53^−/−^* cells transduced with dCas9-HA-NLS-mCherry-Krab-NLS and POT1 variants expressed in the pHAGE2-EF1a-MCS-IRES-blast vector. The experiment was performed following guidelines described before (Gilbert et al. 2014). Briefly, the sgRNA library was transfected into HEK293T cells together with third-generation lentiviral packaging vectors to generate lentivirus, the viral supernatant was collected at 48 h and 72 h, snap-frozen, and stored at −80°C until use. After determining the transduction efficiency in each cell line, a large-scale transduction was performed aiming to achieve a low multiplicity of infection (MOI; achieved MOI of 0.3-0.4 for HT1080, MOI of ∼0.6 for RPE-1 *TP53^−/−^*) and a coverage of >300× of the library. After 2 d from the infection, the cells were selected with puromycin to enrich for the sgRNA integration, then the final representation was determined. HT1080 cells were selected with puromycin 800 ng/mL for 2 d, while the RPE-1 p53-null cells were selected with 20 µg/mL puromycin for 4 d. Following selection, at least 2 × 125 mil. cells were frozen down per condition as time point 0 samples (T0), while the rest of the cells were further cultured, plating the equivalent number of cells to achieve ∼1000× coverage of the library per condition. The cells were split every 2–3 d, aiming to maintain 1000× coverage of the library at plating throughout the screen. HT1080 cells were cultured in maintenance hygromycin (50 µg/mL), while RPE-1 p53-null cells were cultured in maintenance blasticidin (7.5–10 µg/mL) to ensure the exogeneous expression of the POT1 constructs is maintained throughout the screen.

The endpoint was reached after approximately nine population doublings for HT1080 cells and ∼14 population doublings for the RPE-1 p53-null cells. At least 2 × 125 million cells were frozen down per condition at the endpoint. Following genomic extraction, the DNA was digested overnight with ∼400 U/mg SbfI-HF (New England Biolabs) in order to enrich for the cassette containing the sgRNA construct. Subsequently, sgRNAs were amplified by PCR and, during the enrichment, Illumina adapters were added to the 3′ and 5′ end of the sgRNA cassette together with an index barcode at one end. Following PCR cleanup, the samples were sequenced on an Illumina HiSeq-4000 using custom primers, single-end 50 bp with a 6-bp index. For each analyzed condition, we obtained at least ∼50 million total reads per condition.

Custom sequencing primers were as follows 5′ sequencing primer: 5′-GTGTGTTTTGAGACTATAAGTATCCCTTGGAGAACCACCTTGTTG-3′, and 3′ sequencing primer: 5′ TGATAACGGACTAGCCTTATTTAAACTTGCTATGCTGTTTCCAGCTTA-3′.

### Bioinformatic analysis of the CRISPRi screen

Sequencing reads were aligned to the library sequences using a custom Python script (available at https://github.com/mhorlbeck/ScreenProcessing). For the HT1080 analysis the sgRNA read counts were normalized to account for differences in total reads across samples. Then, the normalized reads were averaged across the two replicates per condition at each time point. The averaged read counts were used to compute the fold changes at the endpoint compared with day 0 for all conditions. sgRNAs with <25 reads at the T0 time point were excluded from the fold change calculation. Subsequent analysis of the screen was done using the Bayesian analysis of gene essentiality (BAGEL) algorithm (Hart et al. 2015; Hart and Moffat 2016). Empirically determined reference gene sets, including 687 core essential genes and 927 nonessential genes (Hart et al. 2017) were used to generate log_2_ Bayes factor scores. The BAGEL algorithm was ran using bootstrapping and the network boosting option, which uses the pre-existing information from the functional associations annotated in the STRING v10.5 database (Szklarczyk et al. 2017) to refine the log_2_ Bayes factor scores. Log_2_ Bayes factors (BFs) were also computed for the individual HT1080 replicates, starting from normalized read counts, and using the cross-validation option for BAGEL. The correlation between the HT1080 replicates for each condition was determined with Pearson's coefficient and r-squared analysis. The performance of the screen was determined using the reference sets of essential and nonessential genes to compute precision-recall curves. To determine the different fitness genes between the wild-type and mutant POT1 conditions, a *z*-score reflecting a “differential essentiality” score was computed (Steinhart et al. 2017) for each “essential” gene (BF > 3) in the mutant POT1 condition: For each analyzed gene, the BF score for POT1-WT was subtracted from the BF score for the respective POT1 mutant; *z*-scores were subsequently assigned to each BF scores difference, in order to highlight the most “differential essential” genes between the wild-type and mutant POT1 conditions. The analysis for RPE-1 p53-null cells was done similarly as for HT1080, using the BAGEL algorithm employing cross-validation and the network boosting option.

Pathway enrichment analysis was conducted using PANTHER 14.1 (http://www.pantherdb.org; Mi et al. 2019) with the default statistical overrepresentation test parameters for Reactome pathways (Fabregat et al. 2018).

Data were plotted using matplotlib version 3.1.1.

### Bira*-mediated biotinylation and pull-down of proteins

HT1080 cells stably expressing empty vector, pLPC-N-Myc-FLAG-BirA*-POT1 or pLPC-N-Myc-FLAG-BirA*-POT1-ΔOB were grown in 15-cm^2^ dishes to 90% confluence at the time of harvesting. Approximately 100 million cells per condition were treated with biotin (50 µM) for 16–20 h prior to harvesting. The cells were then collected by trypsinization, pooled, and counted. The cells were then pelleted, washed twice with 1× PBS and resuspended in cold NP40 lysis buffer (10 mM Tris-HCl at pH 7.4, 10 mM NaCl, 3 mM MgCl2, 0.5% NP40 with freshly added protease inhibitors [Roche]). In order to allow for a gentle fractionation, the resuspended cells were incubated rocking for ∼1 h at 4°C, until an enriched nuclear fraction could be observed under the light microscope. The nuclei were pelleted at 3300*g* for 10 min at 4°C, resuspended very well in room temperature SDS lysis buffer (1% SDS, 10 mM EDTA at pH 8, 50 mM Tris-HCl at pH 8, with freshly added 1 mM PMSF, protease inhibitors), and then incubated for 10 min on ice. Next, the samples were boiled 5 min at 95°C. After cooling on ice, the samples were sonicated on Biorupter UCD-200 ice-water bath 10 sec on/ 20 sec off, high setting until they became clear and fluid (at least 10 min). Subsequently, the samples were centrifuged at 13,000 rpm for 10 min at 4°C to clear any debris. The supernatant was diluted 1:10 with IP dilution buffer (1.2 mM EDTA; 16.7 mM Tris-HCl at pH 8; 150 mM NaCl with freshly added 1 mM PMSF and protease inhibitors), loaded on Amicon Ultra 3K columns and spun at 4000*g* for 30 min at 4°C to remove free biotin and concentrate the sample. The concentrate was transferred into fresh tubes and supplemented with Triton X-100 to 1% final concentration. The amount of concentrate used per IP was equalized among samples based on the initial cell number calculated during harvest. After saving ∼5% of lysate as input, the rest was incubated overnight with Dynabeads MyOne Streptavidin C1 (Thermo Fisher 65001**)** at 4°C, rotating end-over-end. The next day, the beads were washed twice in buffer 1 (2% SDS in water) at room temperature, once in buffer 2 (0.1% deoxycholate, 1% Triton X-100, 500 mM NaCl, 1 mM EDTA, 50 mM HEPES at pH7.5) at 4°C, once in buffer 3 (250 mM LiCl, 0.5% NP40, 0.5% deoxycholate, 1 mM EDTA, 10 mM Tris at pH 8.1) at 4°C, and twice in buffer 4 (50 mM Tris-HCl at pH 7.4, 50 mM NaCl) at 4°C. All washes were performed for 8–10 min using an end-over-end rotator. After the last wash, the beads were resuspended in buffer 4 and 4× Laemmli buffer and boiled for 5 min at 95°C to elute the bound proteins. Approximately 5% of the eluate was used for silver stain and Western blot analysis, while the rest was submitted for mass spectrometry. The silver stain was done according to the manufacturer's instructions (Pierce silver stain kit, Thermo Fisher 24612). The experiment was conducted independently three times.

### Preparation of samples for mass spectrometry

The samples were resuspended in NuPAGE LDS sample buffer (Novex). The proteins were reduced with 2 μL of 0.2M dithiothreitol (Sigma) for 1 h at 57°C at pH 7.5. Next, the proteins were alkylated with 2 μL of 0.5M iodoacetamide (Sigma) for 45 min at room temperature in the dark. The samples were loaded on a 1.0-mm NuPAGE® 4%–12% Bis-Tris gel (Life Technologies) and run for 8 min at 200V. The gel was stained with GelCode Blue stain reagent (Thermo). The gel bands were excised, cut into 1-mm^3^ pieces and destained for 15 min in a 1:1 (v/v) solution of methanol and 100 mM ammonium bicarbonate. The buffer was exchanged and the samples were destained for another 15 min. This was repeated for another three cycles. The gel plugs were dehydrated by washing with acetonitrile, and further dried by placing them in a SpeedVac for 20 min. Two-hundred-fifty nanograms of sequencing-grade modified trypsin (Promega) was added directly to the dried gel pieces followed by enough 100 mM ammonium bicarbonate to cover the gel pieces. The gel plugs were allowed to shake at room temperature and digestion proceeded overnight. The digestion was halted by adding a slurry of R2 50 μm Poros beads (Applied Biosystems) in 5% formic acid and 0.2% trifluoroacetic acid (TFA) to each sample at a volume equal to that of the ammonium bicarbonate added for digestion. The samples were allowed to shake for 120 min at 4°C. The beads were loaded onto C18 ziptips (Millipore), equilibrated with 0.1% TFA, using a microcentrifuge at 6,000 rpm for 30 sec. The beads were washed with 0.5% acetic acid. Peptides were eluted with 40% acetonitrile in 0.5% acetic acid, followed by 80% acetonitrile in 0.5% acetic acid. The organic solvent was removed using a SpeedVac concentrator and the sample reconstituted in 0.5% acetic acid.

### Mass spectrometry analysis

An aliquot of each sample was loaded onto an Acclaim PepMap trap column (2 cm × 75 µm) in line with an EASY-Spray analytical column (50 cm × 75 µm ID PepMap C18, 2-μm bead size) using the auto sampler of an EASY-nLC 1000 HPLC (Thermo Fisher Scientific) with solvent A consisting of 2% acetonitrile in 0.5% acetic acid and solvent B consisting of 80% acetonitrile in 0.5% acetic acid. The peptides were gradient eluted into an Orbitrap Elite mass spectrometer (Thermo Fisher Scientific) using the following gradient: 5%–35% in 60 min, 35%–45% in 15 min, followed by 45%–100% in 5 min. The gradient was held at 100% for another 10 min. MS1 spectra were recorded with a resolution of 60,000, an AGC target of 1e6, with a maximum ion time of 200 msec, and a scan range from 400 to 1500 m/z. Following each full MS scan, 15 data-dependent MS/MS spectra were acquired. The MS/MS spectra were collected in the Ion Trap with an AGC target of 3e4, maximum ion time of 150 msec, one microscan, 2 m/z isolation window, dynamic exclusion of 30 sec, and normalized collision energy (NCE) of 35.

### Data processing for mass spectrometry

The MS/MS spectra were searched against the UniProt *Homo sapiens* reference proteome database (downloaded October 2015) using Andromeda (Cox et al. 2011). Protein quantitation was performed using Intensity-based absolute quantification (iBAQ) (Schwanhäusser et al. 2011) within MaxQuant (Cox and Mann 2008) (Version 1.5.3.30). The mass tolerance was set to 20 ppm for MS1. MS2 searches were set to a tolerance of 0.5 Da. False discovery rate (FDR) filtration was done first on peptide level and then on protein level. Both filtrations were done at 1% FDR using a standard target-decoy database approach. All proteins identified with less than two unique peptides were excluded from analysis.

### Guide RNA sequences

Guide RNA sequences used here were as follows: sgA _HUS1: 5′-GAGCCGCGGCGGGCCTCTGT-3′; sgB_HUS1: 5′ GTACCCACAGAGGCCCGCCG-3′; sgC_HUS1: 5′-GGGATGAAGGCGGCTTTCAA-3′; nontargeting-sgA: 5′-GCGTACGACAATACGCGCGA-3′; nontargeting-sgB: 5′-GTTACTACAGGAGCCGAAGG-3′; nontargeting-sgC: 5′-GGCGCCGGACTGGACCTCGA-3′; and nontargeting-sgD: 5′-GGCGCTCCCACCGATAAAGT-3′.

### Cell-Titer-Glo cell viability assay

sgRNAs targeting *HUS1* (A, B, and C) and nontargeting guides RNAs (A, B, C, and D) from the CRISPRi library individually cloned into lentiGuide-puro (Addgene 52963) were used to virally transduce RPE-1 p53-null cells expressing dCas9-HA-NLS-mCherry-Krab-NLS and POT1 variants cloned into pHAGE2-EF1a-MCS-IRES-blast. At 48 h postinfection, the cells were put on puromycin selection (20 µg/mL) to enrich for the sgRNA-expressing cells. Following completion of the puromycin selection, the cells were trypsinized, counted twice, and following serial dilutions, 500 cells were plated in triplicate per condition in 96 wells. The cells were left to grow until they neared confluence in the nontargeting guides wells (8 d for POT1-WT/ POT1-ΔOB; 7 d for POT1-K90E), then cell growth was quantified using CellTiter-Glo (Promega) according to the manufacturer's instructions. The growth was normalized to the average growth in the nontargeting sgRNA-A wells for each cell line (POT1-WT, POT1-K90E, and POT1-ΔOB, respectively).

### shRNA sequences

The shRNAs against *NUP58/NUPL1, NUP62, POLD3, SMC2, TPR, NUP153*, and *HUS1* were obtained from the MISSION shRNA Sigma-Aldrich library. The shRNAs against *TERF2*, namely TRF2 sh-F (Open Biosystems TRCN0000004811) and TRF2 sh-G (Open Biosystems TRCN0000018358), were created in the Salk Institute Gene Transfer, Targeting, and Therapeutics Core or the CMRI Vector and Genome Engineering Facility.

The shRNAs used were as follows: shRNA_NUP58-A (TRCN0000280396): CCGGGCTTCAGTTTAGGATTCAATACTCGAGTATTGAATCCTAAACTGAAGCTTTTTG; shRNA_ NUP58-B (TRCN0000013517): CCGGGCGGCACAACTTCAGTCTATTCTCGAGAATAGACTGAAGTTGTGCCGCTTTTTG; shRNA_NUP62-A (TRCN0000232576): CCGGGCAACCTCACTAATGCCATATCTCGAGATATGGCATTAGTGAGGTTGCTTTTTG; shRNA_NUP62-B (TRCN0000232579): CCGGGCTTTCATTTGAGTATCTTTGCTCGAGCAAAGATACTCAAATGAAAGCTTTTTG; shRNA_POLD3 (TRCN0000233260): CCGGGATAGTGAAGAGGAGCTTAACCTCGAGGTTAAGCTCCTCTTCACTATCTTTTTG; shRNA_SMC2 (TRCN0000291367): CCGGCCAGATTTACTCAATGTCAAACTCGAGTTTGACATTGAGTAAATCTGGTTTTTG; shRNA_TPR (TRCN0000060066): CCGGGCGATCTGAAACAGAAACCAACTCGAGTTGGTTTCTGTTTCAGATCGCTTTTTG; shRNA_NUP153 (TRCN0000290594): CCGGCCCAGTTTCTTTAACTCCATTCTCGAGAATGGAGTTAAAGAAACTGGGTTTTTG; shRNA_ HUS1 (TRCN0000439497): CCGGGCGCACAGTCAGTCTTG CATTCTCGAGAATGCAAGACTGACTGTGCGCTTTTTG; shRNA_TRF2 (TRCN0000004811, sh-F): CCGGGCGCATGACAATAAGCAGATTCTCGAGAATCTGCTTATTGTCATGCGCTTTTTG; and shRNA TRF2 (TRCN0000018358, sh-G): CCGGGACAGAAGCAGTGGTCGAATCCTCGAGGATTCGACCACTGCTTCTGTCTTTTTG; and scramble control hairpin sequence (a gift from David Sabatini, sequence from plasmid Addgene 1864): CCTAAGGTTAAGTCGCCCTCGCTCGAGCGAGGGCGACTTAACCTTAGG.

### Incucyte S3 live-cell analysis

RPE-1 p53-null cells expressing either POT1-WT or POT1-ΔOB cloned into pHAGE2-EF1a-MCS-IRES-blast were transduced with pLKO.1-puro scramble control or shRNAs targeting the genes of interest. For the siRNA-mediated depletion of WAPAL, the cells were transfected with siRNA (WAPL siRNA: GGUUAAGUGUUCCUCUUAUdTdT [Dharmacon]; control siRNA:ON-TARGETplus nontargeting control pool [Horizon Discovery]) using Lipofectamine RNAiMAX (Thermo Fisher Scientific) according to the manufacturer's instructions. Following puromycin selection for shRNAs or at 24 h posttransfection with siRNA, 25,000 cells per condition were plated in 12 wells and 16 fields were imaged per well every 8 h using the 10× objective on the Incucyte S3 live-cell analysis system (Essen Bioscience, Inc.). When the control cells neared confluence, we used the Incucyte analysis software with the default settings and the 1.2 segmentation adjustment to determine the percentage of confluence over time in each well. For the cleanup step, the hole fill was set to 0 and no adjustments were made to the pixel size. No area or eccentricity filters were applied. At least two independent experiments were performed with duplicate wells for all shRNA-related experiments. The siRNA-mediated WAPAL depletion experiment was performed once with triplicate wells.

### Mitotic DNA synthesis (MiDAS) detection and telomeric FISH

Detection of mitotic DNA synthesis on metaphase plates was performed according to the experimental procedures for detecting MiDAS on metaphase chromosomes published by (Garribba et al. 2018). To note, HT1080 cells expressing exogenous POT1 variants were not synchronized or treated with aphidicolin for MiDAS. HT1080 cells were incubated with 20 µM EdU and 100 ng/mL colcemid (Roche) for 75 min to arrest cells in metaphase. RPE-1 p53-null cells expressing exogenous POT1 variants were treated with 0.4 µM aphidicolin for 40 h. In the last 16 h of the aphidicolin treatment the RPE-1 p53-null cells were concurrently treated with 9 µM RO3306 to enrich for cells in G2/M. The RPE-1 p53-null cells were washed three times with 1×PBS to release them from arrest, then incubated for 50–60 min at 37°C with 20 µM EdU and 100 ng/mL colcemid (Roche). Following metaphase arrest, the cells were harvested, incubated in freshly made hypotonic 0.075 M KCl solution for 20–30 min at 37°C, followed by an overnight fixation in cold methanol/acetic acid (3:1). Metaphase spreads were generated by dropping cells onto prechilled glass slides. EdU detection was performed with a Click-IT EdU Alexa flour 594 imaging kit (Life Technologies), as described previously (Garribba et al. 2018). Telomeric FISH was performed as previously described (Sfeir et al. 2009) using an Alexa488-labeled TelC PNA probe (PNA Bio, Inc.). The cell cycle profiles of untreated versus aphidicolin and RO3306 treated RPE-1 p53-null cells were determined using propidium iodide staining.

### Detection of ultrafine anaphase bridges

Ultrafine anaphase bridges in U2OS osteosarcoma cells exogenously expressing wild-type POT1 and POT1-ΔOB were detected using the mouse monoclonal anti-PICH antibody (clone 14226-3; 1:100 dilution; Millipore 04-1540), by following the coextraction protocol described by Bizard et al. (2018)

### Live-cell imaging

RPE-1 p53-null cells stably expressing exogenous POT1 variants and H2B-GFP were plated on uncoated 35-mm glass-bottom microwell dishes (MatTek, no. 1.5 coverglass P35G-1.5-14-C) and incubated overnight. Approximately 6 h before imaging, the media was replaced with a 1:10 ratio of regular cell culture medium to live-cell imaging medium (Live-Cell Imaging Solution, Life Technologies A14291DJ) supplemented with 10% FBS, 2 mM L-glutamine, 100 U/mL penicillin, 0.1 μg/mL streptomycin, 0.1 mM nonessential amino acids, 1 mM sodium pyruvate (Life Technologies 11360070) and 20 mM dextrose (Sigma D9434). One experiment was imaged on a Nikon Eclipse Ti total internal reflection fluorescence/epifluorescence inverted microscope fitted with an Andor Zyla camera, using a Plan Fluor 40× DIC M N2 objective, 100 ms exposure and 2 × 2 binning. Two subsequent, independent experiments were imaged on a Nikon Eclipse Ti inverted microscope equipped with an Andor Neo camera, using a Plan Fluor 40× DIC M N2 objective, 220-msec exposure and 2 × 2 binning, at 7% intensity of the 488-nm laser. Both microscopes were equipped with environmental chambers and automated stages. All experiments were imaged for 15 h at 2-min intervals, capturing nine z-stacks with 1.2-µm steps. Movies were analyzed using the Nikon NIS-Elements software. Mitotic duration was determined between nuclear envelope breakdown and anaphase. Counts were blinded.

### IF-FISH

IF-FISH experiments were performed as described before (Takai et al. 2003). The primary antibodies used were 53BP1 (1:1000; rabbit polyclonal; Novus Biologicals 100-304A) and Myc (1:1000; Cell Signaling 9B11). The secondary antibodies used were raised against rabbit or mouse and conjugated with Alexa 488 (Invitrogen) or Rhodamine Red-X (Invitrogen), and diluted 1:1000. Telomere FISH was performed using Alexa 488 or Cy3-OO-TelC-labeled PNA probes (PNA Bio, Inc.). Streptavidin Alexa 488 **(**1:2000 dilution; Invitrogen S32354, ) was used for detection of biotinylated proteins by IF.

### Western blots

Cells were lysed in lysis buffer (50 mM Tris-HCl at pH 7.4, 150 mM NaCl, 1 mM EDTA, 0.1% SDS, 1% Triton-X, 1 mM DTT, 1 mM PMSF, supplemented with complete protease inhibitor mix [Roche]) or in RIPA buffer supplemented fresh with complete protease inhibitor mix for 30 min on ice, followed by centrifugation at maximum speed for 10 min at 4°C. An equal volume of 4× Laemmli buffer was added to the supernatants and the samples were boiled for 5 min. Approximately 1 × 10^5^ cells or between 15 and 30 µg of protein were separated on an SDS-PAGE gel, followed by blotting to nitrocellulose membranes. The membranes were blocked in 5% BSA in TBST or in 5% milk in TBST, incubated with primary antibodies, followed by an incubation with horseradish peroxidase (HRP) secondary antibodies (mouse IgG HRP-linked whole Ab, 1:5000 [GE HealthcareNA931]; rabbit IgG HRPlinked whole Ab, 1:5000 [GE Healthcare NA934]) in 3% milk in TBST. Primary antibodies were as follows: M2 Flag (1:10,000 dilution; Sigma Aldrich), anti-c-Myc antibody clone 9E10 (1:1000 dilution; Millipore), anti-HA.11 clone 16B12 (1:1000 dilution; Biolegend, previously Covance MMS-101P, ), γ-tubulin clone GTU-88 (1:5000 dilution; Sigma Aldrich), WAPL (1:10,000 dilution; Abcam ab109537), TPR (1:1000 dilution; Abcam ab84516), NUP153 (1:1000 dilution; Abcam ab24700), SMC2 (1:1000 dilution; Abcam ab10412), POLD3 (1:1000 dilution; Abcam ab182564), GAPDH (1:10,000 dilution; Bio-Rad 12004167), rabbit polyclonal Arp2 (1:1000; Cell Signaling Technology 3128), mouse monoclonal Arp3 (1:1000; Abcam ab49671), rabbit anti-TRF2 (Novus Biologicals NB110-57130), anti-NUP62 (dilution 1:100; Sigma HPA039360), and anti-NUP58 (1:200; Santa CruzBiotechnology sc-48373,). Streptavidin-HRP (Invitrogen 1953050) was used for detection of biotinylated proteins.

### Superresolution imaging of telomeres and F-actin filaments in S phase

Forty-eight hours after transfection of the NLS-GFP-actin and RFP-PCNA chromobodies and 24 h after addition of aphidicolin or Latrunculin B (LatB; Cayman Chemical 10010631), cells were fixed in 3.7% formaldehyde/1× PBS for 10 min, then permeabilized with 0.5% Triton/1× PBS and blocked with 5% bovine serum albumin (Sigma-Aldrich)/1× PBS. Slides were incubated for 3 h at 37°C with polyclonal anti-TRF2 antibody, washed four times for 5 min in 1× PBS, then incubated for 1 h at 37°C with goat polyclonal anti-rabbit Alexa Fluor 647. First and secondary antibodies were diluted in 1× PBS supplemented with 5% FCS. Before mounting, slides were washed again as described above and dehydrated in a graded series (70%, 90%, and 100%) of ethanol solutions for 2 min each. Slides were then mounted with Prolong Gold (Life Technologies) and imaged using superresolution microscopy.

For siRNA mediated inhibition of the ARP2/3 complex experiments, the cells were transfected with the NLS-GFP-actin and RFP-PCNA chromobodies, with control nontargeting (control siRNA, D-001810-10) or ACTR2 (L-012076-5) and ACTR3 (L-012077-5) ON-TARGETplus siRNA pools (Dharmacon) using DharmaFECT 1 Transfection Reagent (Dharmacon), according to the manufacturer's protocol. Cells were fixed and immunostained 72 h posttransfection.

Superresolution imaging was performed on a Zeiss LSM 880 AxioObserver confocal fluorescent microscope fitted with an Airyscan detector using a plan-apochromat 63× 1.4 NA M27 oil objective. Cells were imaged using 1.9% excitation power of 568-nm laser, 2% excitation power of 488-nm laser, and 1.8% excitation power of 647-nm laser, with 1 × 1 binning for all laser conditions in combination with the appropriate filter sets. Ten or more z stacks (167 nm) were captured with frame-scanning mode and unidirectional scanning. Z-stacks were Airyscan processed using batch mode in Zen software.

Imaging data was imported into Imaris 8.4.1 software, where nuclei were segmented as “cells” using the Cell function on the actin-NLS channel. Telomeres were segmented as “vesicles” using the “cell” function. The distance between foci and the nuclear periphery were calculated using the function “distance of vesicles to cell membrane.”

### CO-FISH analysis

The CO-FISH was performed as previously described (Sfeir et al. 2009), based on the de Lange laboratory protocol (http://delangelab.rockefeller.edu/assets/file/FISH%20COFISH%20protocol.pdf), with the following modifications: The slides were stained with 1 µg/mL Hoechst 33258 (Sigma) in 2× SSC for 15 min at room temperature, followed by exposure to 365-nm UV for 10.8 × 10^3^ J/m^2^ and subsequent digestion with 1600 units Exonuclease III (Promega) for 30 min at 37°C. Telomere FISH was performed using an Alexa 488 TelG PNA probe (1:1000 dilution; PNA Bio Inc.) overnight, followed hybridization with the Cy3-OO-TelC labeled PNA probe (1:1000 dilution; PNA Bio Inc.) for 2 h at room temperature.

### Generation of cells with POT1 knock-in mutations using CRISPR/Cas9

The RPE-1 *POT1^K90E/K90E^* clone was generated using CRISPR/Cas9 as previously described (Pinzaru et al. 2016). The RPE-1 *POT1^F62V/+^* knock-in was generated using the Cas9 nickase and the guide RNAs 5′- GTTACAACTGAGCAATAATC-3′ and 5′- CTAACTTGCCTGCTCTTTAG-3′, cloned in the pSpCas9n(Bbs)(Bsa)-2A-GFP plasmid (PX461; Addgene plasmid 48140). A double-stranded template with ∼500-bp homology arms, carrying the F62V mutation and a silent mutation creating a SpeI site for genotyping was used as a donor template. The RPE-1 cells were transfected with the Cas9 nickase–sgRNAs construct and the donor template using Lipofectamine 3000 (Thermo Fisher Scientific) according to the manufacturer's protocol, then GFP-positive single cells were flow sorted into 96 wells 48 h after transfection. The ensuing clones were genotyped by SpeI digestion of a PCR product flanking the targeted region. Successful RPE-1 *POT1^F62V/+^* targeting was confirmed by Sanger sequencing of the genomic region (using primers amplifying outside of the donor template sequence) and the *POT1* cDNA.

### Compteting interest statement

Agnel Sfeir is a cofounder, consultant, and shareholder in Repare Therapeutics.

## Supplementary Material

Supplemental Material
