# Supplementary material for: Replication stress conferred by POT1 dysfunction promotes telomere relocalization to the nuclear pore
Source: Genes Dev. 2020 Dec 1;34(23-24):1619–36. doi: 10.1101/gad.337287.120 (PMC7706707; doi:10.1101/gad.337287.120)
Supplement: Supplemental Material [file supp_34_23-24_1619__index.html]

Replication stress conferred by POT1 dysfunction promotes telomere relocalization to the nuclear pore — Supplemental Material 

# Replication stress conferred by POT1 dysfunction promotes telomere relocalization to the nuclear pore

## Supplemental Material

- Supplementary\_Table\_S1.xlsx
- Supplementary\_Table\_S2.xlsx
- Supplemental\_Table\_S3.xlsx
- Supplementary\_Table\_S4.xlsx
- Supplemental\_Figures\_and\_Tables\_AP.pdf
