# Supplementary material for: Replication stress conferred by POT1 dysfunction promotes telomere relocalization to the nuclear pore
Source: Genes Dev. 2020 Dec 1;34(23-24):1619–36. doi: 10.1101/gad.337287.120 (PMC7706707; doi:10.1101/gad.337287.120)
Supplement: Supplemental Material [file supp_gad.337287.120_Supplemental_Figures_and_Tables_AP.pdf]

Supplemental Figures and Tables:

Figure S1

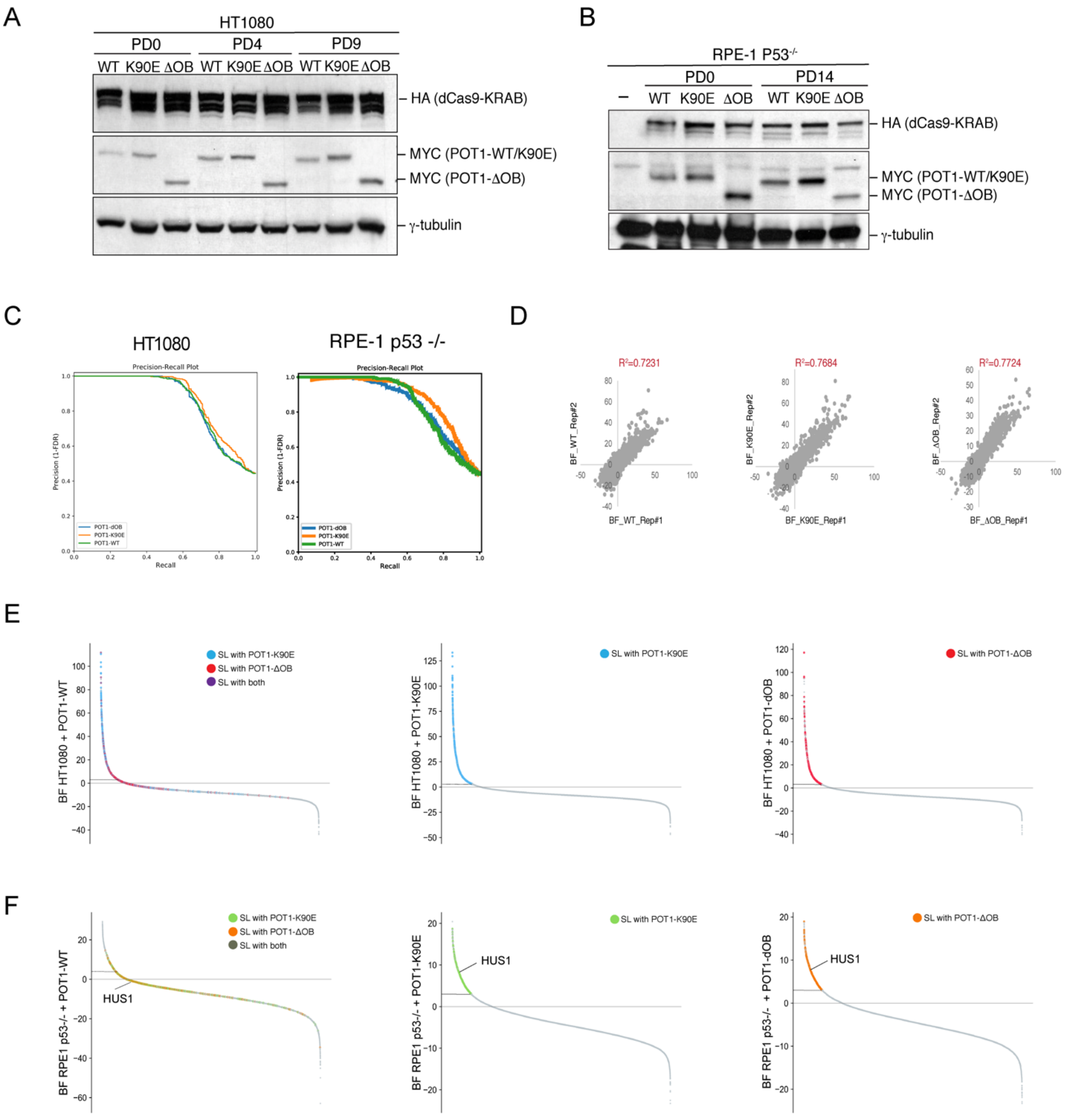

Figure S1 related to Figure 1: Genome-wide CRISPR interference screen analysis in cells expressing wild-type or *POT1* OB-fold mutations. (A – B) Western blot analysis monitoring expression of the catalytically inactive dCas9-KRAB fusion (dCas9-KRAB) and POT1 constructs in cells at initial and final time points of the screens. Western blot for HT1080 in (A) and RPE-1

$p53^{-/-}$ in (B). **(C)** Precision-recall curves for the BAGEL analysis of the genome-wide screens: the average of the two HT1080 replicates (left) and RPE-1 p53 $^{-/-}$ screen (right). (**D)** Correlation of BF factors between the replicates for the HT1080 screen for each POT1 variant. Rep#1 – replicate 1; Rep#2 – replicate 2. **E)** Ranked log Bayes Factor (BF) scores computed using BAGEL for each screen condition in HT1080 cells. Genes are represented on the x-axis and Bayes Factor scores on the y-axis. The dotted horizontal line represents a threshold of BF $\geq$ 3 for 'essential' genes. Highlighted dots represent SL candidate genes: blue, SL with POT1-K90E; red, SL with POT1-ΔOB; dark purple, SL candidate with both POT1-K90E and POT1-ΔOB. **(F)** Similar analysis as in (E) for the RPE1 p53-/- screen. Highlighted dots represent SL candidate genes: green, SL with POT1-K90E; orange, SL with POT1-ΔOB; maroon, SL candidate with both POT1-K90E and POT1-ΔOB.

Figure S2

A

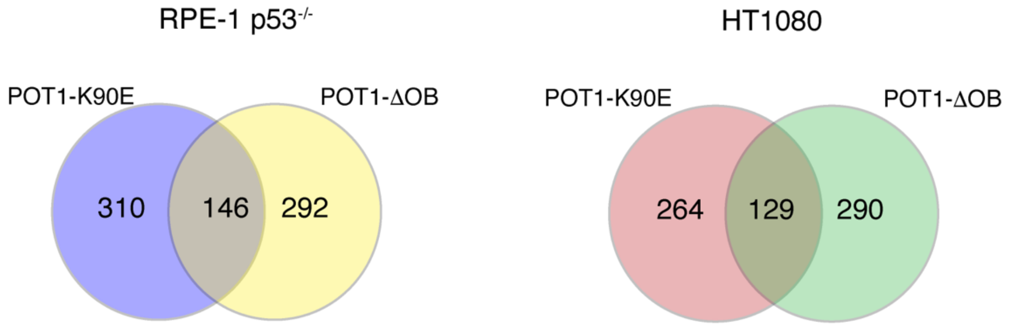

B

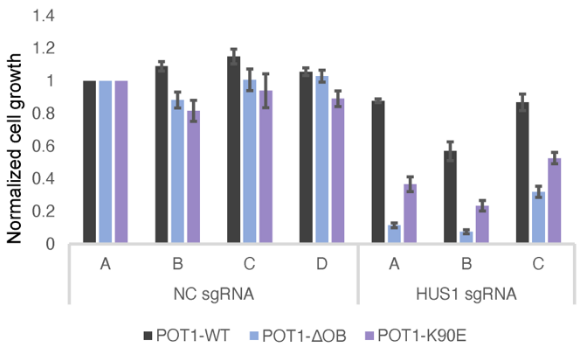

C

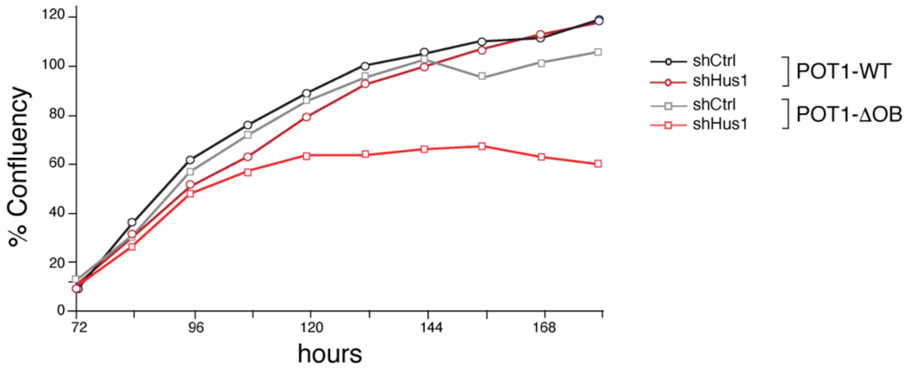

Figure S2 related to Figure 1: Analysis of synthetic lethality candidates with *POT1* OB-fold mutations. (A) Venn diagrams illustrating the overlap between the synthetic lethality (SL) candidates with the two *POT1* OB-fold mutations in RPE-1 p53$^{-/-}$ (left) and HT1080 (right) cells. **(B)** CellTiter-Glo analysis of cell growth in RPE1 p53$^{-/-}$ expressing dCas9-KRAB and the indicated POT1 constructs, following transduction with 4 different non-targeting sgRNAs (NC) and 3 different sgRNAs targeting HUS1. Cells were plated in triplicate in 96 wells (500 cells/well) following puromycin selection for the sgRNAs. Cell growth was assessed with CellTiter-Glo. The growth rate was normalized to the wells expressing NC sgRNA-A for each POT1 construct. Plotted is the mean $\pm$ SD across triplicates. **(C)** Incucyte survival assay of RPE-1 p53$^{-/-}$ expressing the indicated POT1 constructs following transduction with non-targeting shRNA and shRNAs targeting *HUS1*. Cells were plated in triplicate for each treatment.

Figure S3

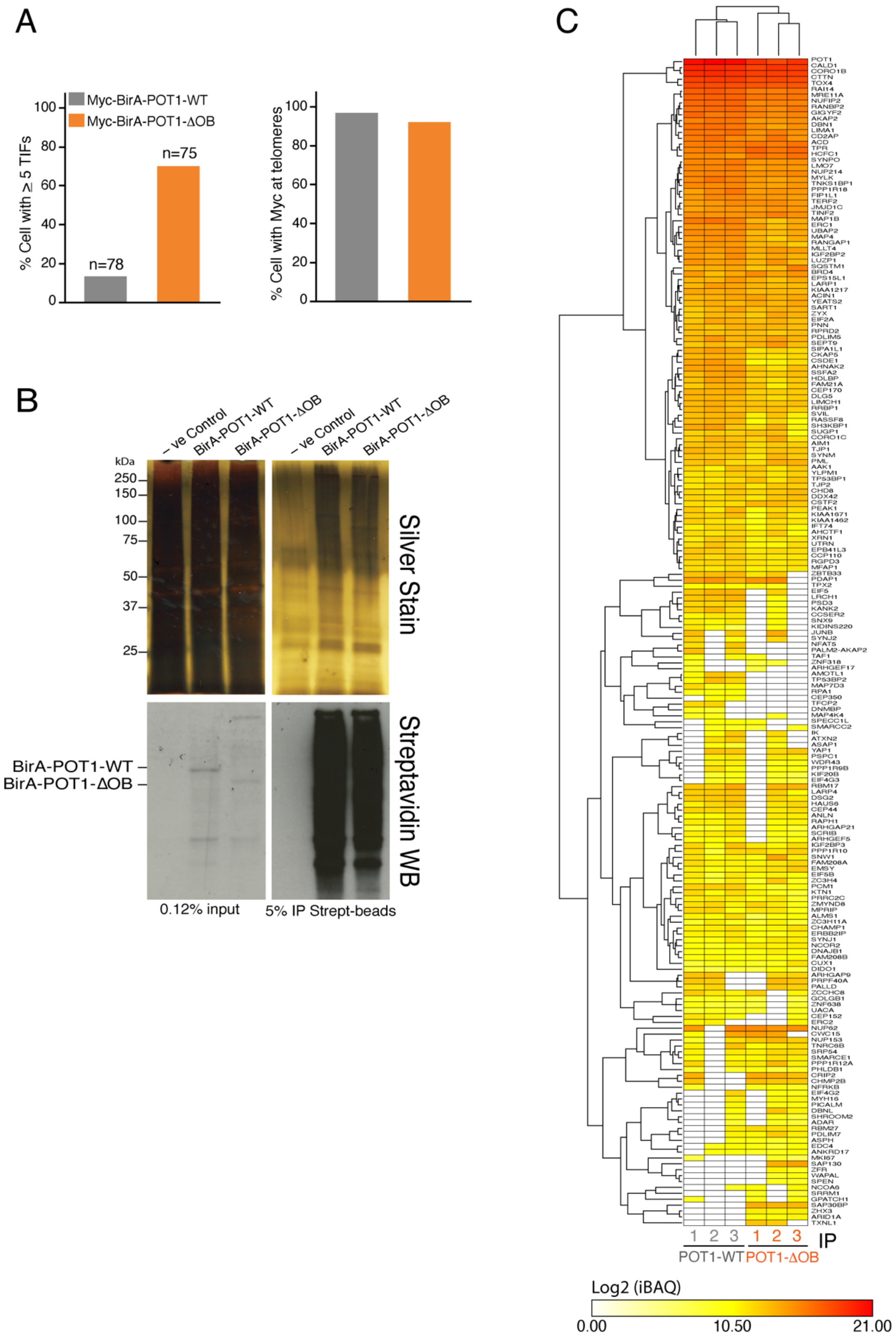

A
Myc-BirA-POT1-WT
Myc-BirA-POT1-ΔOB
% Cell with ≥ 5 TIFs
n=78
n=75
% Cell with Myc at telomeres
0
20
40
60
80
100
B
– ve Control
BirA-POT1-WT
BirA-POT1-ΔOB
kDa
250
150
100
75
50
37
25
Silver Stain
Streptavidin WB
BirA-POT1-WT
BirA-POT1-ΔOB
0.12% input
5% IP Strept-beads
C
1
2
3
IP
POT1-WT
POT1-ΔOB
Log2 (iBAQ)
0.00
10.50
21.00

Figure S3 related to Figure 2: BirA*-mediated biotinylation and pull down of proteins in HT1080 cells expressing wild-type or an OB-fold mutated POT1. (A) (Left) Quantification of telomere dysfunction-induced foci (TIFs) in HT1080 cells expressing Myc-tagged BirA*-POT1-WT and BirA*-POT1-ΔOB. (Right) Quantification of cells with Myc co-localization at telomeres in HT1080 cells expressing the indicated constructs. **(B)** Representative silver stain (top) and streptavidin Western blot (bottom) from a streptavidin immunoprecipitation (IP) in the indicated cells. **(C)** Unsupervised hierarchical clustering of the log2-transformed intensity-base absolute quantification (iBAQ) values for proteins recovered in at least 2 IPs per condition. Included proteins do not appear in the negative control IPs and are represented in less than 25% of IPs documented in the CRAPome database (Mellacheruvu et al. 2013). Clustering was done using complete Euclidean distance using the Morpheus software (https://software.broadinstitute.org/morpheus).

Figure S4

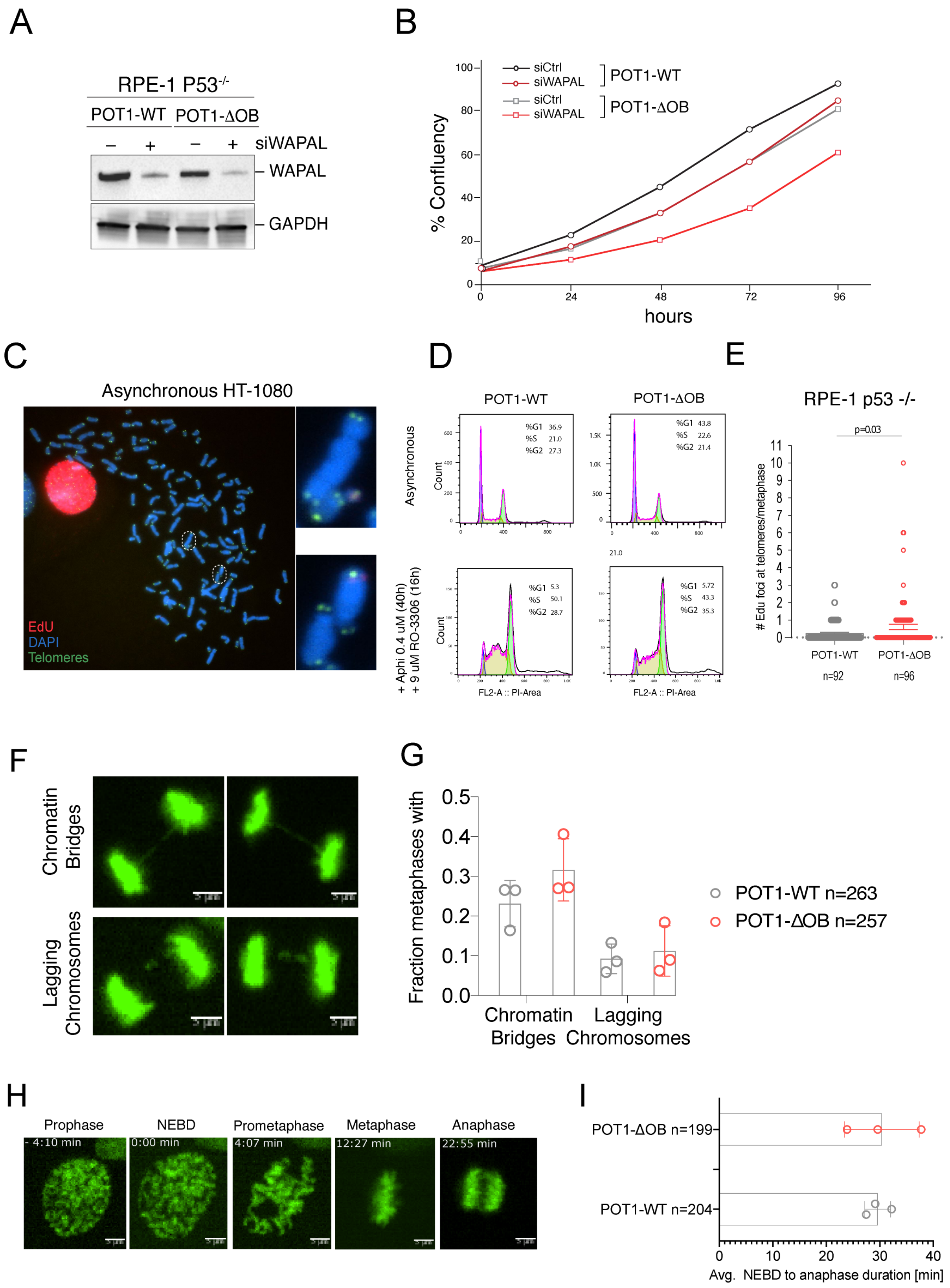
A
RPE-1 P53-/-
POT1-WT
POT1-ΔOB
siWAPAL
WAPAL
GAPDH
B
siCtrl
siWAPAL
POT1-WT
siCtrl
siWAPAL
POT1-ΔOB
% Confluency
hours
C
Asynchronous HT-1080
EdU
DAPI
Telomeres
D
POT1-WT
POT1-ΔOB
Asynchronous
+ Aphi 0.4 uM (40h)
+ 9 uM RO-3306 (16h)
Count
FL2-A :: PI-Area
E
RPE-1 p53 -/-
p=0.03
# Edu foci at telomeres/metaphase
POT1-WT
POT1-ΔOB
n=92
n=96
F
Chromatin Bridges
Lagging Chromosomes
G
Fraction metaphases with
Chromatin Bridges
Lagging Chromosomes
POT1-WT n=263
POT1-ΔOB n=257
H
Prophase
NEBD
Prometaphase
Metaphase
Anaphase
- 4:10 min
0:00 min
4:07 min
12:27 min
22:55 min
I
POT1-ΔOB n=199
POT1-WT n=204
Avg. NEBD to anaphase duration [min]

Figure S4 related to Figure 3: Analysis of mitoses in cells expressing wild-type or *POT1* OB-fold mutations. (A) Validation of growth defect upon inhibition of MiDAS factor WAPAL in cells expressing POT1-WT and POT1-ΔOB. **(**A) Western blot analysis for WAPAL. (B) Graph depicting cellular growth monitored by Incucyte in the indicated cells. **(C)** Representative image for EdU incorporation at telomeres in asynchronous HT1080 cells. Insets show enlarged chromosomes with EdU staining (red) at telomeres (green). DNA is stained by DAPI in blue. **(D)** Cell cycle profile of asynchronous (top) and aphidicolin +RO-3306 treated (bottom) RPE-1 $p53^{-/-}$ cells expressing POT1-WT and POT1-ΔOB. DNA was stained using propidium iodide (PI-area) (x-axis) and quantified using flow cytometry. **(E)** Quantification of experiments in Fig. 3F, examining the number of EdU foci at telomeres per metaphase. p-value was determined using the unpaired, non-parametric Mann-Whitney test. **(F, G)** Analysis of chromosome aberrations in mitosis using live-cell imaging of RPE1 $p53^{-/-}$ cells expressing the indicated POT1 constructs and labeled with H2B-GFP. (F) Representative images of chromatin bridges and lagging chromosomes. Scale bar is set at 5 µm. (G) Quantification of the metaphases with chromatin bridges or lagging chromosomes (including micronuclei).Bars represent mean $\pm$ SD of three independent experiments. The total number of analyzed mitoses per condition is indicated on the right. **(H, I)** Analysis of the duration from nuclear envelope breakdown to anaphase onset using live-cell imaging of RPE1 $p53^{-/-}$ cells expressing the indicated POT1 constructs and labeled with H2B-GFP. (H) Representative images of the mitotic stages, from prophase to anaphase onset. Scale bar is set at 5 µm. (I) Quantification of the average duration of mitosis for cells expressing each of the indicated POT1 constructs. Bars represent the average of 3 independent experiments $\pm$ SD; total number of analyzed mitoses per condition is indicated on the graph.

Figure S5

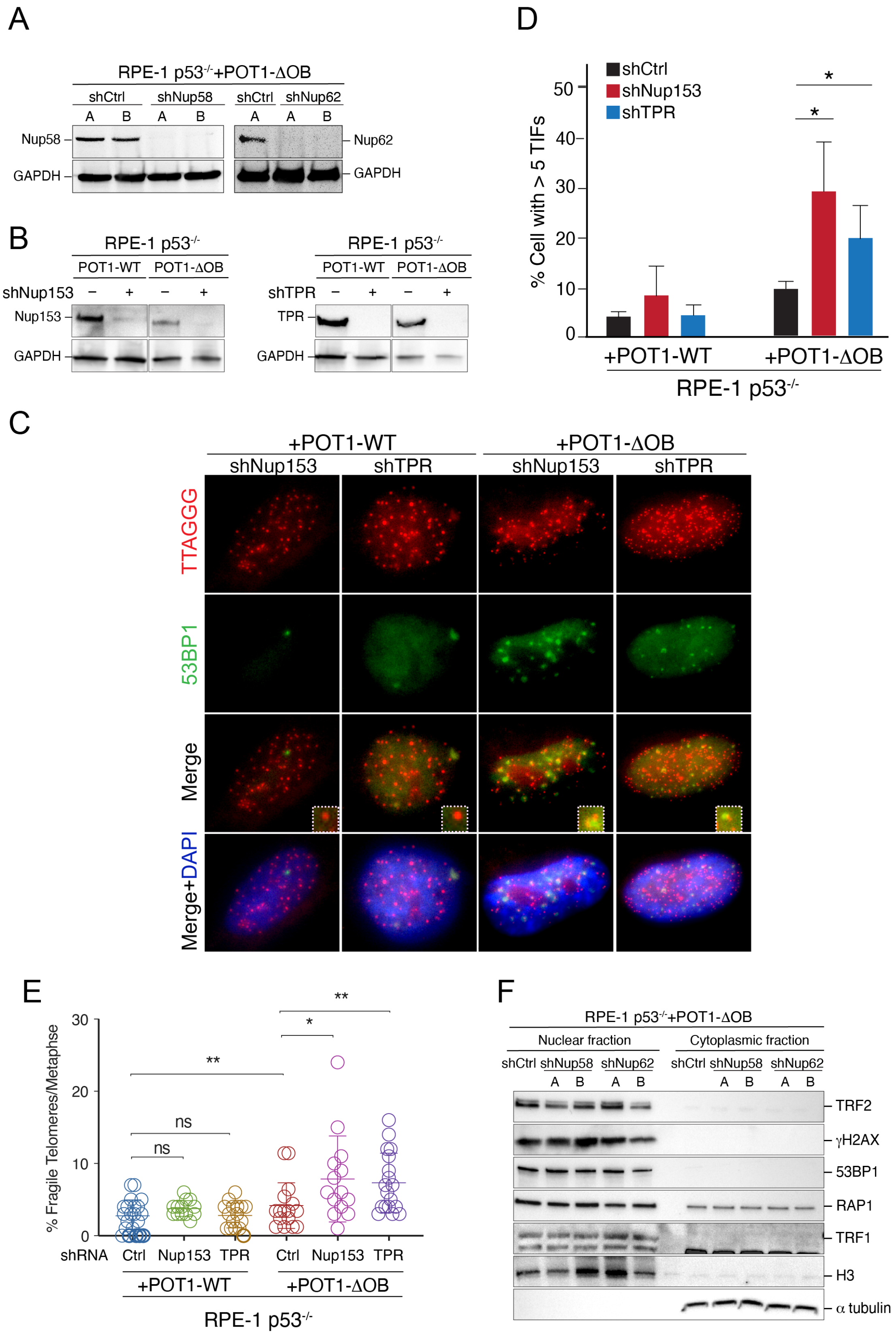

A
RPE-1 p53-/-+POT1-ΔOB
shCtrl shNup58 shCtrl shNup62
A B A B A A B
Nup58
GAPDH
Nup62
GAPDH
B
RPE-1 p53-/-
POT1-WT POT1-ΔOB
shNup153 – + – +
Nup153
GAPDH
RPE-1 p53-/-
POT1-WT POT1-ΔOB
shTPR – + – +
TPR
GAPDH
C
+POT1-WT +POT1-ΔOB
shNup153 shTPR shNup153 shTPR
TTAGGG
53BP1
Merge
Merge+DAPI
D
shCtrl
shNup153
shTPR
% Cell with > 5 TIFs
0 10 20 30 40 50
*
*
+POT1-WT +POT1-ΔOB
RPE-1 p53-/-
E
% Fragile Telomeres/Metaphse
0 10 20 30
**
*
**
ns
ns
shRNA Ctrl Nup153 TPR Ctrl Nup153 TPR
+POT1-WT +POT1-ΔOB
RPE-1 p53-/-
F
RPE-1 p53-/-+POT1-ΔOB
Nuclear fraction Cytoplasmic fraction
shCtrl shNup58 shNup62 shCtrl shNup58 shNup62
A B A B A B A B
TRF2
γH2AX
53BP1
RAP1
TRF1
H3
α tubulin

Figure S5 related to Figure 4: shRNA-mediated depletion of NUPs in cells expressing exogenous POT1 constructs. (A) Western blot showing shRNA-mediated depletion of NUP62 and NUP58 in RPE1 p53 $^{-/-}$ cells expressing POT1-ΔOB and POT1-WT. **(B)** Western blot showing shRNA-mediated depletion of NUP153 and TPR in the indicated cells. **(C)** Representative images displaying telomere dysfunction-induced foci (TIFs) in RPE-1 p53$^{-/-}$ cells expressing POT1-ΔOB and treated with shRNAs against NUP153 and TPR as well as control shRNA. 53BP1 in green is detected by indirect immunofluorescence, and telomeres are marked with FISH in red. DNA is counterstained with DAPI in blue. **(D)** Quantification of the percentage of cells with 5 or more TIFs in cells with the indicated treatment. Graph represents the mean of n=3 independent experiments with SD (two-tailed t-test). **(E)** Analysis of telomere fragility in RPE-1 p53$^{-/-}$ cells expressing POT1-WT and POT1-ΔOB and treated with the indicated shRNA. Graph representing quantification of fragile telomeres per metaphase in cells with the indicated treatments with SD (one -way ANOVA test). **(F)** Fractionation experiment in RPE-1 p53$^{-/-}$ cells treated with shNUP58 and shNUP62, and control shRNA. Western blot depicts efficient nuclear import of TRF1, TRF2, RAP1, 53BP1 and γ-H2AX. Anti-H3 antibody marks nuclear protein. α-tubulin marks cytoplasmic fractions.

Figure S6

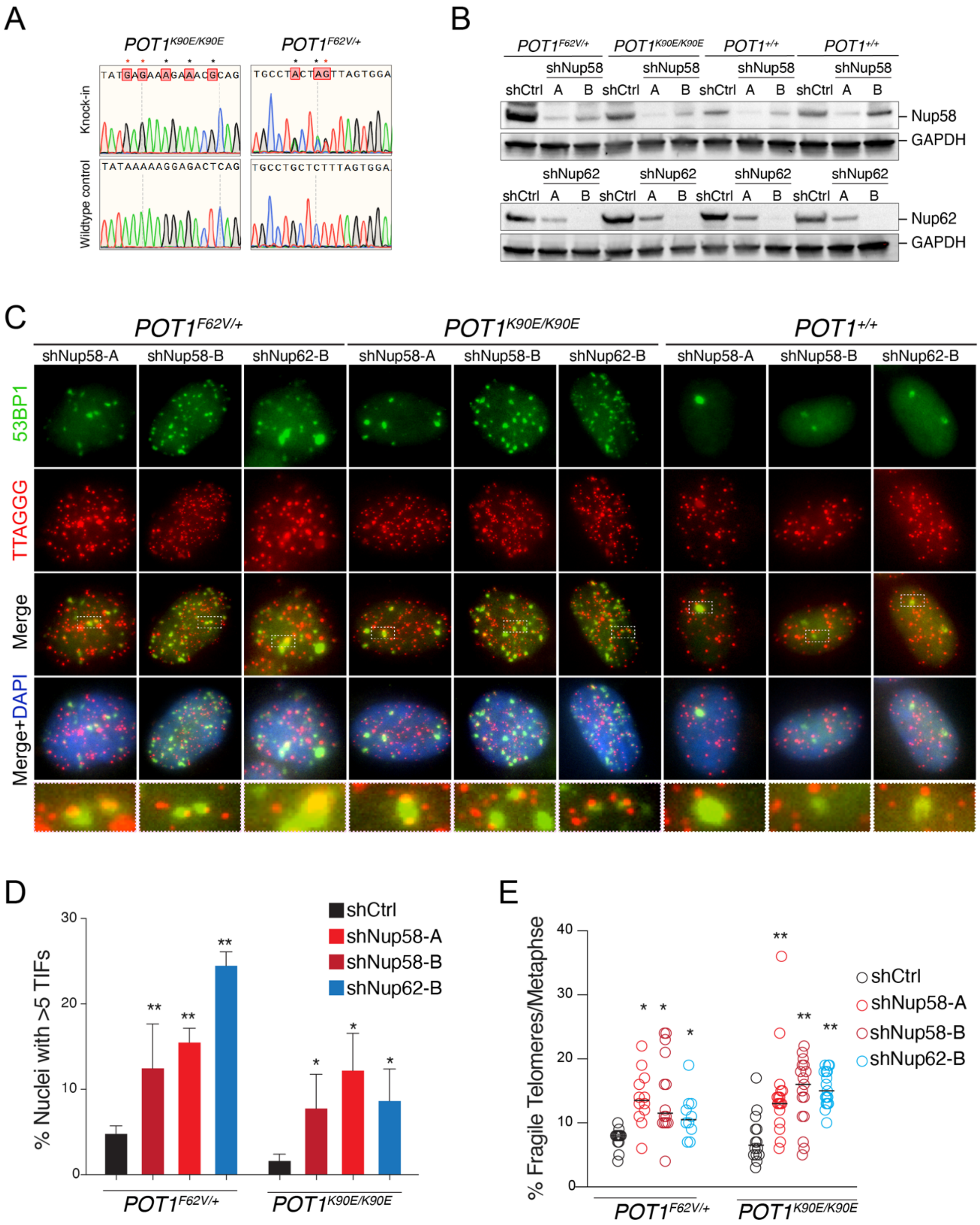

Figure S6 related to Figure 4: shRNA-mediated depletion of NUP58 and NUP62 in cells with knock-in *POT1* mutations. (A) Sanger sequencing confirming gene editing with CRISPR-Cas9. Red stars denote the codon mutation of interest; black stars are silent mutations for genotyping. The sequences are in frame, showing codon triplets. (**B)** Western blot showing shRNA-mediated depletion of NUP62 and NUP58 in cells with the indicated genotype. **(C)** Representative images

displaying telomere dysfunction-induced foci (TIFs) in cells with the indicated genotype treated with shRNAs against NUPs as well as control shRNA. 53BP1 in green is detected by indirect immunofluorescence, and telomeres are marked with FISH in red. DNA is counterstained with DAPI in blue. **(D)** Quantification of the percentage of cells with 5 or more TIFs in the targeted cells depleted from the indicated NUPs. Graph represents the mean of n=3 independent experiments with SD (two-tailed t-test). **(E)** Analysis of telomere fragility in the indicated cells. Graph representing quantification of fragile telomeres per metaphase in cells with the indicated treatments with SD (one -way ANNOVA test).

Figure S7

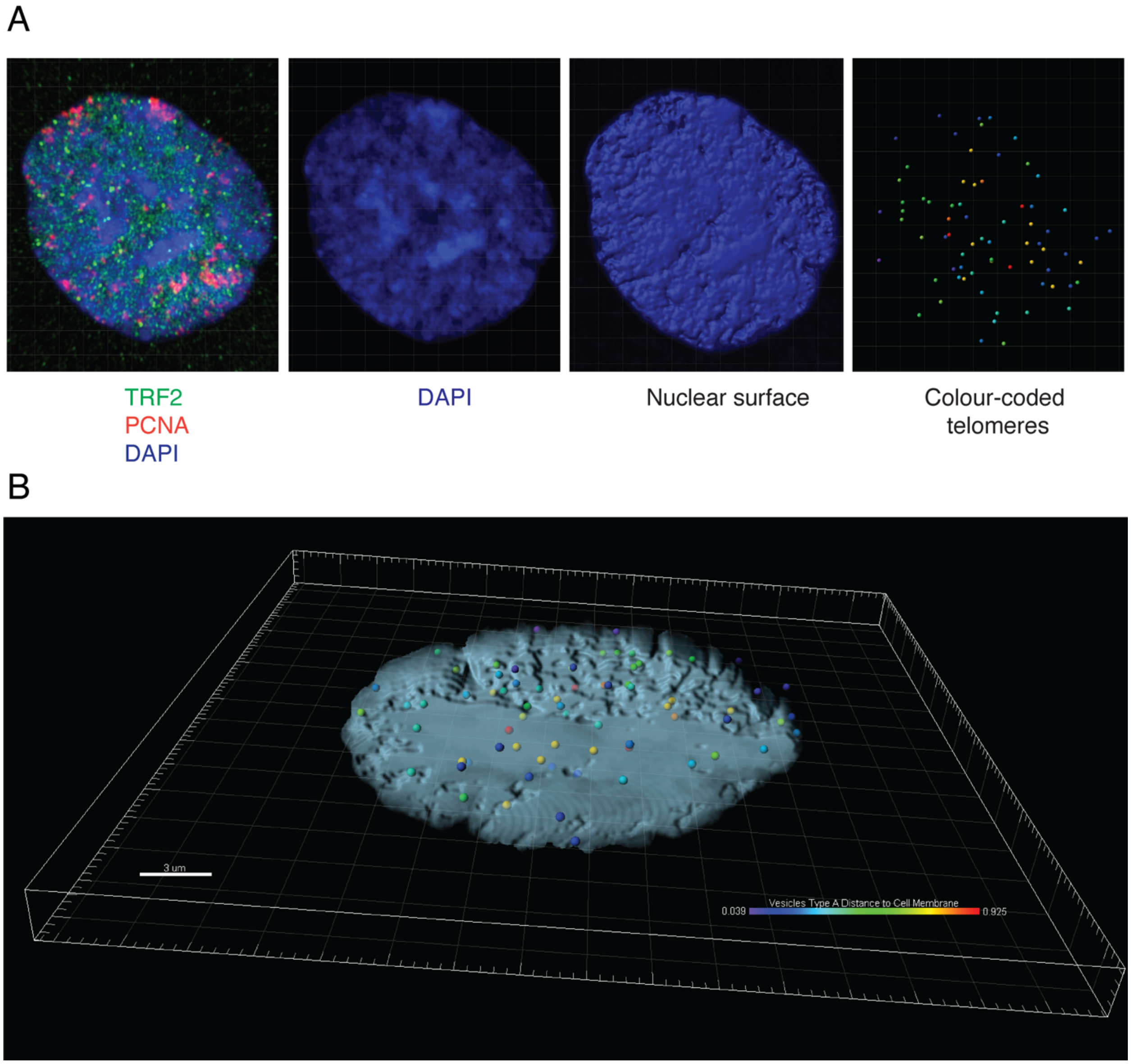

Figure S7 Related to Figure 5: Analysis of telomere localization in the nucleus. (A) Representative super-resolution microscopy of a single Z plane taken from a three-dimensional image through the nuclear volume of fixed cells stained with NLS-GFPactin and RFP-PCNA chromobodies and stained with DAPI and TRF2 to mark telomeres. (B) TRF2 foci were identified

throughout the three-dimensional volume of the image in (A) and their distances from nuclear periphery identified. In the analysis, three-dimensional images consisting of at least 12 Z-planes separated by 0.185 μm were acquired. The distance from the nuclear periphery in three-dimensions was then attained for each telomere. These data were compressed into a maximum projection rendering in which each telomere is color-coded for its respective distance from the nuclear periphery.

Figure S8

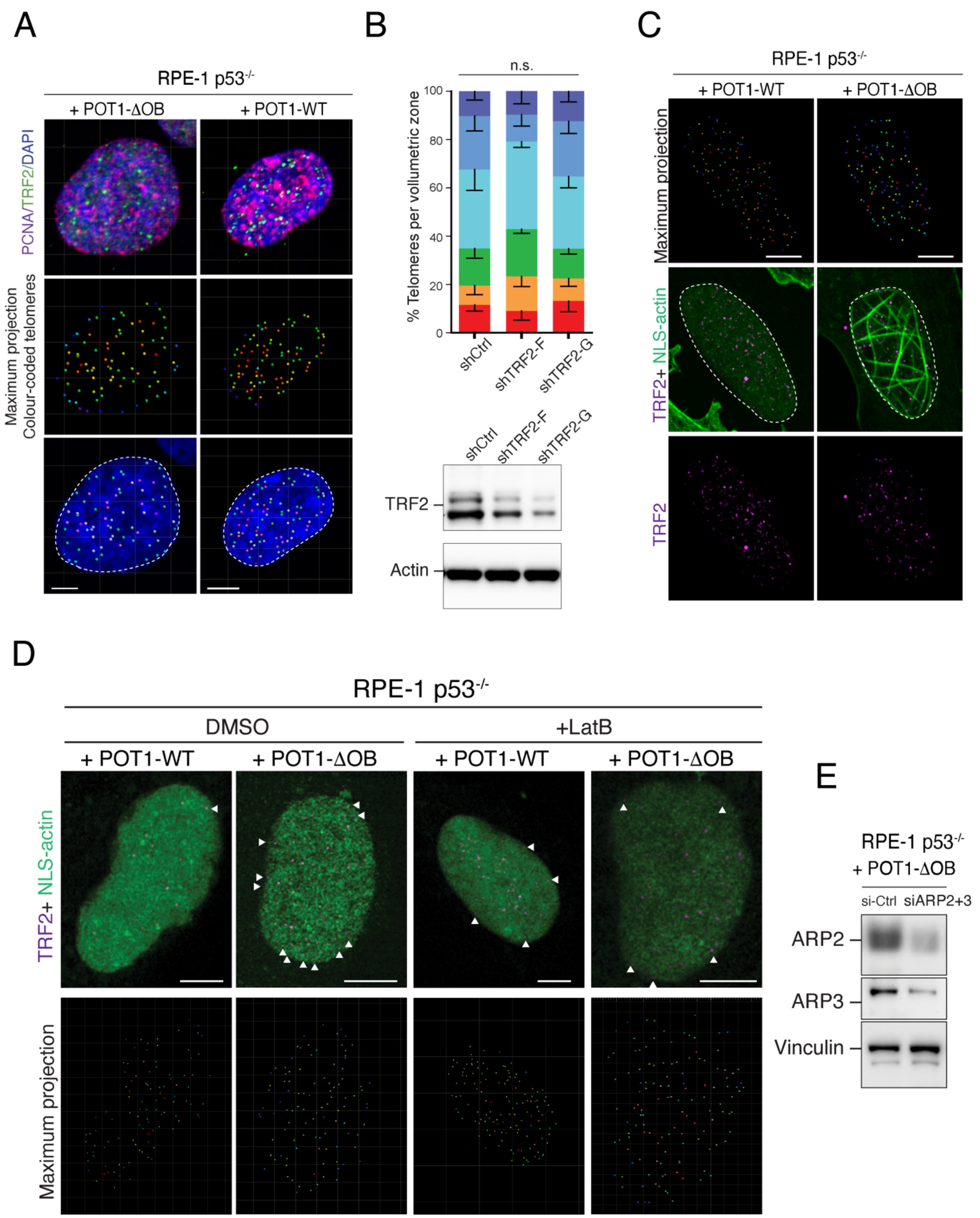

A
RPE-1 p53-/-
+ POT1-ΔOB
+ POT1-WT
PCNA/TRF2/DAPI
Maximum projection
Colour-coded telomeres
B
n.s.
% Telomeres per volumetric zone
100
80
60
40
20
0
shCtrl
shTRF2-F
shTRF2-G
TRF2
Actin
C
RPE-1 p53-/-
+ POT1-WT
+ POT1-ΔOB
Maximum projection
TRF2+ NLS-actin
TRF2
D
RPE-1 p53-/-
DMSO
+LatB
+ POT1-WT
+ POT1-ΔOB
TRF2+ NLS-actin
Maximum projection
E
RPE-1 p53-/-
+ POT1-ΔOB
si-Ctrl
siARP2+3
ARP2
ARP3
Vinculin

Figure S8 related to Figure 5: F-actin filament formation in cells expressing a *POT1* OB-fold mutation. (A) Representative super-resolution microscopy of a three-dimensional (3D) image through the nuclear volume of fixed RPE-1 p53$^{-/-}$ cells expressing POT1-WT and POT1-ΔOB. S-phase cells were marked with PCNA (red) and telomeres detected with an anti-TRF2 antibody (magenta). Cells were counterstained with DAPI (blue) to mark nuclear periphery. **(B)** (top) Quantification of telomere localization in the nucleus of cells with two independent shTRF2 and control shRNA. Graph represents distribution of telomeres with respect to the nuclear periphery. Mean ± SEM (chi-square test). (bottom) Western blot analysis with anti-TRF2 antibody in cells treated with shTRF2 and control shRNA. **(C)** Representative super-resolution microscopy of a three-dimensional (3D) image through the nuclear volume of fixed cells exogenously expressing POT1-WT or POT1-ΔOB. Telomeres were detected with an anti-TRF2 antibody. The cells were transfected with an NLS-GFP-Actin chromobody 48h prior to fixation. **(D)** Representative super-resolution microscopy of three-dimensional (3D) images through the nuclear volume of fixed RPE-1 p53$^{-/-}$ cells exogenously expressing POT1-WT and POT1-ΔOB. The cells were transfected with an NLS-GFP-Actin chromobody 48 h prior to fixation, and either with DMSO or Latrunculin B (LatB) for 24h prior to fixation. Telomeres were detected with an anti-TRF2 antibody. The arrowheads indicate telomeres at the nuclear periphery. **(E)** Western blot analysis with the indicated antibody in cells treated with siARP2, siARP3, and control siRNA.

Figure S9

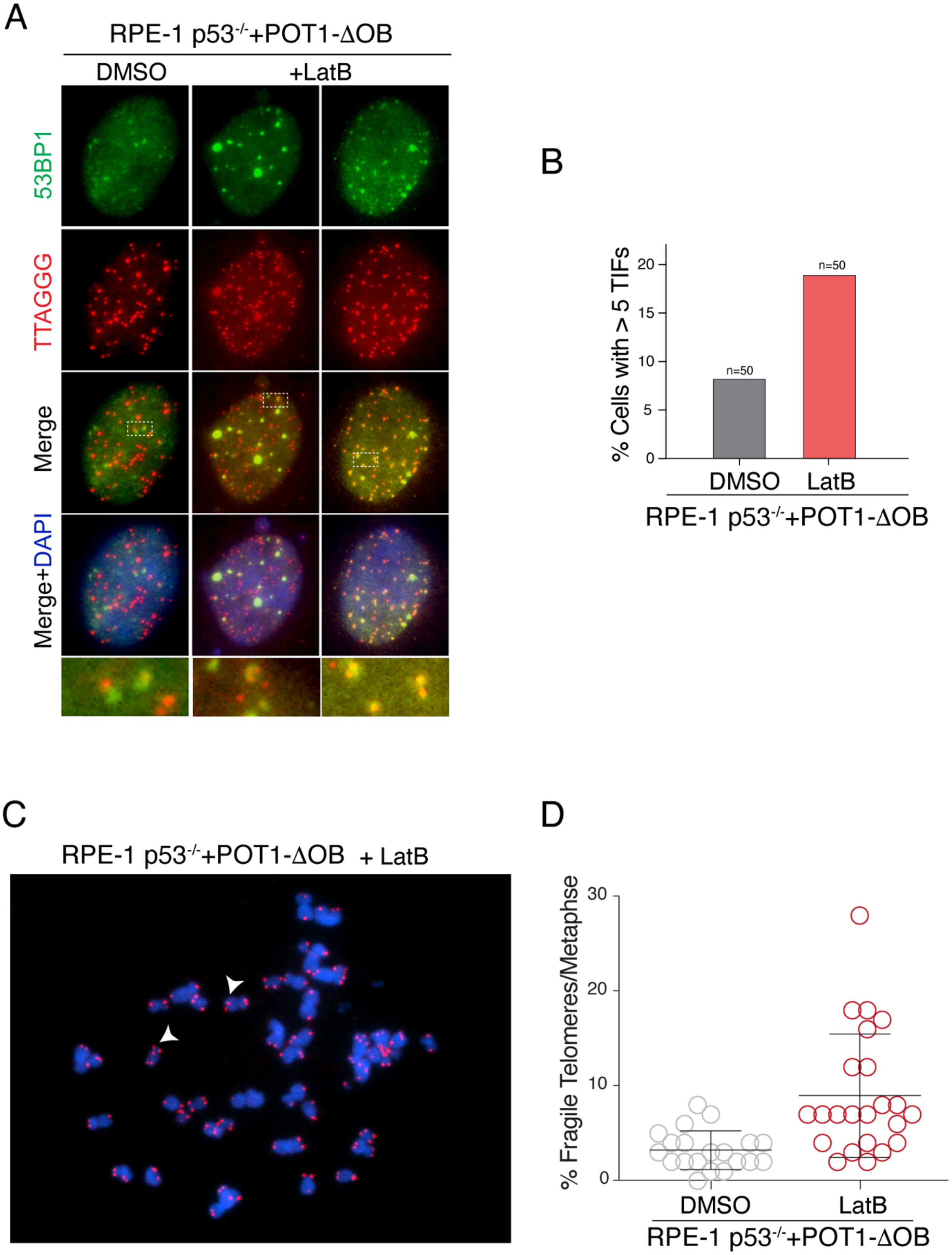

A
RPE-1 p53-/-+POT1-ΔOB
DMSO
+LatB
53BP1
TTAGGG
Merge
Merge+DAPI
B
% Cells with > 5 TIFs
n=50
n=50
DMSO
LatB
RPE-1 p53-/-+POT1-ΔOB
C
RPE-1 p53-/-+POT1-ΔOB + LatB
D
% Fragile Telomeres/Metaphse
DMSO
LatB
RPE-1 p53-/-+POT1-ΔOB

Figure S9 related to Figure 5: Inhibition of F-actin filament formation augments telomere fragility and dysfunction in cells expressing a *POT1* OB-fold mutation. (A) Telomere dysfunction-induced foci (TIFs) in RPE-1 $p53^{-/-}$ cells expressing POT1-ΔOB and treated with LatB and DMSO control. 53BP1 in green is detected by indirect immunofluorescence, and telomeres are marked with FISH in red. DNA is counterstained with DAPI in blue. **(B)** Quantification of the percentage of cells with 5 or more TIFs in cells with the indicated treatment. **(C)** Analysis of telomere fragility in RPE-1 $p53^{-/-}$ cells expressing POT1-ΔOB and treated with LatB and DMSO control. Graph representing quantification of fragile telomeres per metaphase in cells with the indicated treatments.

Supplemental Table S1: BAGEL analysis BF scores for the genome-wide screens and 'differential essentiality' z-scores.

Supplemental Table S2: Comparison of BF scores for the HT1080 individual replicates.

Supplemental Table S3: Pathway over-representation analysis of the synthetic lethality candidates using PANTHER.

Supplemental Table S4: Analysis of the iBAQ values for the proteins recovered from the BioID experiments in HT1080 cells expressing BirA*-POT1-WT or BirA*-POT1- ΔOB.
